# Making strides in doctoral-level career outcomes reporting: a review of classification and visualization methodologies in graduate education

**DOI:** 10.3389/feduc.2025.1462887

**Published:** 2025-05-23

**Authors:** Tammy R. L. Collins, Rebekah L. Layton, Deepti Ramadoss, Jennifer MacDonald, Ryan Wheeler, Adriana Bankston, C. Abby Stayart, Yi Hao, Jacqueline N. Robinson-Hamm, Melanie Sinche, Scott Burghart, Aleshia Carlsen-Bryan, Pallavi Eswara, Heather Krasna, Hong Xu, Mackenzie Sullivan

**Affiliations:** 1 National Institutes of Health, National Institute of Environmental Health Sciences, Durham, NC, United States; 2 Burroughs Wellcome Fund, Durham, NC, United States; 3 University of Pittsburgh, Pittsburgh, PA, United States; 4 University of North Carolina at Chapel Hill, Chapel Hill, NC, United States; 5 The New School, New York, NY, United States; 6 The Scripps Research Institute, La Jolla, CA, United States; 7 Future of Research, Pittsfield, MA, United States; 8 University of Chicago, Chicago, IL, United States; 9 University of Maryland, College Park, MD, United States; 10 Federation of American Societies for Experimental Biology, Rockville, MD, United States; 11 University of Virginia, Charlottesville, VA, United States; 12 The University of Texas at Austin, Austin, TX, United States; 13 Massachusetts Institute of Technology, Cambridge, MA, United States; 14 Boston University, Boston, MA, United States; 15 Columbia University Mailman School of Public Health, New York, NY, United States; 16 Maastricht University, Maastricht, Netherlands; 17 University of Minnesota, Minneapolis, MN, United States

**Keywords:** graduate education, doctoral training, program evaluation, career outcomes, taxonomy, PhD

## Abstract

The recent movement underscoring the importance of career taxonomies has helped usher in a new era of transparency in PhD career outcomes. The convergence of discipline-specific organizational movements, interdisciplinary collaborations, and federal initiatives has helped to increase PhD career outcomes tracking and reporting. Transparent and publicly available PhD career outcomes are being used by institutions to attract top applicants, as prospective graduate students are factoring in these outcomes when deciding on the program and institution in which to enroll for their PhD studies. Given the increasing trend to track PhD career outcomes, the number of institutional efforts and supporting offices for these studies have increased, as has the variety of methods being used to classify and report/visualize outcomes. This report comprehensively synthesizes existing PhD career taxonomy tools, resources, and visualization options to help catalyze and empower institutions to develop and publish their own PhD career outcomes. Similar fields between taxonomies were mapped to create a new crosswalk tool, thereby serving as an empirical review of the career outcome tracking systems available. Moreover, this work spotlights organizations, consortia, and funding agencies that are steering policy changes toward greater transparency in PhD career outcomes reporting. Such transparency not only attracts top talent to universities, but also propels research progress and technological innovation forward. Therefore, university administrators must be well-versed in government policies that may impact their PhD students. Engaging with government relations offices and establishing dialogues with policymakers are crucial steps toward staying informed about relevant legislation and advocating for more resources. For instance, much of the recent science legislation in the U.S. Congress, including the Creating Helpful Incentives to Produce Semiconductors (CHIPS) and Science Act, significantly impacts federal agency programs influencing universities. To ensure sustained development, it is imperative to support initiatives that enhance transparency, both in terms of legislation and resources. Increased funding for programs supporting transparency will aid legislatures and institutions in staying informed and responsive. Many efforts presented in this publication have received support from federal and state governments or philantrophic sources, underscoring the need for multifaceted support to initiate and perpetuate this level of systemic change.

## 1 Introduction

In the past decade, there have been growing calls to action for institutions to collect and disseminate career outcomes data for graduate students and postdocs, and to develop common standards for reporting these data (Allum et al., 2014; National Academy of Sciences, National Academy of Engineering, Institute of Medicine, 2014; National Academies of Sciences, Engineering, and Medicine, 2018a,b; Silva et al., 2016; Blank et al., 2017; Mathur et al., 2018a) including the National Institutes of Health Biomedical Research Workforce Working Group Report 2012 (Tilghman et al., 2012). These calls are linked to broad systemic issues that are well-documented (Alberts et al., 2014), including a highly competitive faculty job market with far fewer available positions relative to the supply of PhDs, compensation and training length concerns for postdoctoral scholars, and changing educational and career interests of PhDs.

Numerous efforts and approaches to address the need for better career outcomes data collection have emerged, many of which are described in this report. Efforts coalesce into three major approaches: building coalitions, updating funding obligations, and promoting transparent career outcomes. First, the formation of coalitions of stakeholder working groups or institutions committed to common standards has created purpose-driven communities of thought and action. These groups have clarified the central issues and concentrated the call to action, exemplified by the creation and adoption of the Unified Career Outcomes Taxonomy (UCOT) (Mathur et al., 2018a). Some examples of these groups include Rescuing Biomedical Research (RBR)^1^, the National Institutes of Health Broadening Experiences in Scientific Training Consortium (NIH-BEST)^2^, the Coalition for Next Generation Life Science (CNGLS)^3^, and topically-focused meetings such as the Future Of Bioscience Graduate and Postdoctoral Training conference (FOBGAPT 1 & FOBGAPT 2).^4^ A second set of efforts have focused on updating prerequisites to funding to require the collection and dissemination of institutional outcomes data, such as the National Institute of General Medical Sciences’ Request for Applications (NIGMS RFA) requirements for T32 Training Grants. An increasingly common third approach has focused on the development and implementation of institute- or discipline-specific practices for publicly sharing outcomes data, exemplified by recent activities from the American Historical Association (AHA)^5^ and the National Institute of Environmental Health Sciences (Xu et al., 2018) (NIEHS)^6^, thereby changing the standard expectation for other professional societies and institutions. These efforts to collect, assess, and publish career outcomes of PhD graduates are becoming standard practice and carry significant benefit to institutions. Internally, the data can be used to inform curricular, training, budgetary, benchmarking, and recruitment priorities, while current and prospective trainees might use the data to make informed strategic decisions about their career choices and preparation. On the scale of the global workforce, transparent and standardized reporting of career outcomes data clarifies PhDs’ prevalence and impact on society.

Our aim is to decrease the barriers for institutions to collect and report on the career outcomes for their graduate students by reviewing the various options and resources for undertaking these important tasks, and highlighting their key features so that informed decisions can be made about which tools best suit a particular institution’s needs. This manuscript describes institutions and groups with clearly defined or widely used taxonomies or classification systems. To showcase taxonometric commonalities, we synthesized the extensive options and information collected by different organizations using different classifications. We present a crosswalk tool^7^ that highlights common career classification themes among similar fields across all of the taxonomies examined, including a detailed visualization and explanation of how they are mapped onto each other. This furthers the field by allowing for common comparisons across the many types of career outcomes tracked in doctoral and postdoctoral career outcomes data.

### 1.1 Taxonomy scope

We conducted a review of graduate career outcomes taxonomies that have been either used, developed, or published within the past 10 years. Preference for inclusion was given to taxonomies that met one of the following criteria: (1) widely used at a national level; (2) developed by consensus with multiple stakeholders, including those at professional societies; or (3) those with clearly defined categories and rubrics that facilitate reproducibility. This resulted in the identification of 13 taxonomies, which were developed by governmental organizations, professional societies, Universities and Consortia of institutions, etc. Preference was also given for taxonomies that contained classification of doctoral-level career outcomes (either developed specifically for them or used for doctoral populations), while some may also have applicability to those with Master’s degrees. Descriptions and examples of each taxonomy in action were collected and described herein.

The systems are loosely ordered and grouped based upon whether one builds on another, and whether: (1) it is a nationally-based survey; (2) it was developed by an individual institution or consortium; or (3) it was created by a professional association. We showcase every system’s development and features, with the intention to provide comparable information about each approach. Different data visualization methods are also described with the same intention. In the discussion, we review common themes and differences among approaches and systems, and compare and contrast key parts of each system to assist readers in deciding the classification system that best meets their institutional needs. In considering these needs, institutions may also consider how these data can be used to influence policymakers to continue funding this work through programs at relevant federal agencies – potentially expanding beyond R1s to smaller/different types of universities where these data are needed, but current funding levels and research support may preclude their ability to generate it. For example, in the U.S., funding federal programs that support data collection at Historically Black Colleges and Universities (HBCUs) and Minority Serving Institutions (MSIs) could assist agencies and Congress in diversifying the research pipeline by showcasing examples of graduates who have made an impact in society, thereby encouraging more students and trainees to enter research careers (Panchanathan, 2023).

To assist with understanding relationships between taxonomies, we created a taxonomy crosswalk tool^7^; we mapped relative equivalencies between the taxonomies described herein as well as the classification methodologies of 17 additional groups, including public and private universities, consortia, or professional societies. In mapping these taxonomies, a number of challenges occurred. For example, some taxonomies were too comprehensive to fully map within the tool developed (e.g., there were nearly 1,500 categories to choose from), and these omissions were noted within the tool. Some categories had a tally higher than the total number of taxonomies examined because they were present in multiple ways within a single taxonomy (e.g., tenure-track faculty may have appeared as a variety of different professor job titles). Additionally, some categories were repeated for the purposes of alignment; an asterisk (*) was used to indicate when this “one-to-many” mapping occurred. Another key challenge is that no two taxonomies have categories that are 100% equivalent. This was especially apparent when examining employment categorization between different countries. Nevertheless, efforts were made to ascertain the fundamental meaning of each data field in order to best highlight approximate equivalencies between taxonomies. Furthermore, in order to prevent the loss of granularity when aligning taxonomies that are more complex, multiple rows are depicted back-to-back with the same color to highlight categories that are related.

## 2 Systems for classifying career outcomes: national survey-based systems

Nationally developed systems have the benefit of more standardization, wider adoption, and reporting requirements via the federal government who will benefit from the collected data showcased in this publication. Note, however, that national systems are often slow to adapt to new workforce trends or training innovations unless mandated by the government through legislation or a federal program. For example, current trends in job market changes due to the proliferation of Artificial Intelligence (AI) may not be reflected for some time – whereas non-standard measures may be more flexible to adapt (Clark, 2023).

### 2.1 National science foundation survey of earned doctorates

Since 1957, the National Science Foundation (NSF) has administered an annual census-type survey to all research doctorate earners from accredited U.S. institutions, entitled the Survey of Earned Doctorates (SED)^8^. Data are reported at the end of each calendar year following the survey administration date. This survey is administered using a cross-sectional design to capture information about graduate training and education, and includes information about career outcomes. Development of this tool was sponsored by the National Center for Science and Engineering Statistics (NCSES)^9^ within the NSF, along with multiple federal organizations, including the National Institutes of Health (NIH), Department of Education, and the National Endowment for the Humanities, to provide national-level data and reports on outcomes of doctoral training. The SED provides an annual snapshot of the first destinations of doctoral degree recipients.

The SED contains information about educational and training history, and asks graduates to choose from a set of options regarding what best describes their post PhD graduation plans. The NSF’s SED logic tree asks doctorates to first select broad definitions of their job types, then to choose the sector, and finally to select or describe work activities. Job types to choose from are limited to six (e.g., postdoc or other training position, employed other than postdoc, further education, etc.). Job sectors to choose from are limited to four – education, government, private or nonprofit, or other, further defined by specific descriptions of the place of work (e.g., “Education”: US 4-year college or university, US medical school, etc., “Government”: US federal government, foreign government, etc.) Graduates are also asked to classify primary and secondary work activities into the following – research and development, teaching, management or administration, professional services, or other. To summarize, the main career-related categorization tools used in this survey are first, Job Type; second, Job Sector; and third, Primary and Secondary Work Activities.

With annual survey deployment, the SED provides a large dataset for longitudinal comparisons of first-destinations for doctoral degree holders. Educational history questions allow for longitudinal tracking of the educational path to the doctorate. Data gathered on financial support shows trends of how doctoral students are supported during graduate school and debt levels related to undergraduate and graduate education. Broad data fields can be further broken down by factors such as field of study and sex. Doctoral recipients are surveyed directly.

Executive reports are professionally prepared in easy-to-read, high-level summaries at regular intervals by NSF. All information is available to download as Excel files or PDFs, and some information is visualized in the prepared reports in bar or line-graph format.^10^

### 2.2 NSF survey of doctorate recipients

The Survey of Doctorate Recipients (SDR)^11^ is administered every 2 years and was developed to capture long-term career trajectories of doctoral degree holders from a science, engineering, or health field. This survey, conducted by the NCSES and the NIH, has been conducted biennially since 1973 and is administered to a sample of doctorate recipients from U.S. accredited institutions until they reach the age of 76. Survey data collected via this mechanism focuses more specifically on career pathways taken by science, engineering, and health doctorate holders over time.

The SDR collects data on current employment status and occupational information by asking graduates to specify job responsibilities, and their employers’ main business or industry. Employment sectors are categorized further (e.g., self-employed or business owner, private sector employee, U.S. government employee, or other). Educational institution options are surveyed separately, followed by questions regarding the educational institution and academic position. The SDR continues by asking respondents to account for work activities typically engaged in, selected from a list (e.g., “Accounting…, Basic Research, Applied Research,” etc.). Respondents are also asked to categorize their jobs based on a list that is updated periodically. The list of job categories is further divided into specific occupations within each category (e.g., *Job Category*: “Biological/Life Scientist” is broken down into more specific occupations, including “Biochemists and biophysicists,” etc.). The main categorization tools in this survey are Employment Sector, Work Activities, and Job Category/Occupation.

The NSF’s longstanding history of administering these surveys allows for standardized, longitudinal data collection that enables comparison of trends over time across large, comprehensive data sets. The job categories within the SDR are based off of the Standard Occupational Classification system (SOC; the coding scheme for occupations, US Bureau of Labor Statistics^12^), thus tying into a robust, tested system that is widely used as a standard for classifying careers. The SDR includes granular information about higher education roles (e.g., type of institution, faculty rank, tenure-status, etc.) and also captures over a dozen work activities that occupy at least 10% of the respondent’s time on the job. Additional granular data addresses primary and secondary work activities, type and location of employer, and basic annual salary. The taxonomic categories tracked are fairly broad regarding job titles, but multiple functions can be indicated. Doctoral degree holders are surveyed directly.

The NSF publishes InfoBriefs on employment among the doctoral scientists and engineers, based on the SDR. All information is available to download as Excel files or PDFs, and some information is visualized in the prepared reports in bar or line-graph format.^13^ SDR data are also available to analyze via a special tool termed the ‘Scientists and Engineers Statistical Data System’ (SESTAT). SDR data tables allow for breakdown beyond the major findings in the executive summary and report which focus more on employment status and time to degree (and the intersection with citizenship/international status, gender, etc.), rather than position, title, sector, etc. A multitude of specialized reports analyzing and visualizing various characteristics of the workforce are also available.

### 2.3 Council of graduate schools PhD career pathways

The Council of Graduate Schools (CGS)^14^ initiated the PhD Career Pathways project^15^ as a multi-phase partnership with a coalition of 75 doctoral institutions and involves collecting information on career outcomes by administering a survey. The CGS Alumni Survey contains questions related to career outcomes, and is inspired by the NSF’s SDR^11^ taxonomy described above. Broad categorization tools include three main categories. The first category is Employment Sector (e.g., Education, Government), with sectors further subdivided based on their characteristics (e.g., Education: research university, liberal arts college; Government: US federal, US state or local, etc.). The second category is Job Type (e.g., administrator, faculty member, postdoctoral researcher, etc.). The third category reported is Work Activities (e.g., managing projects, teaching).

The CGS subcategories for educational institutions differ from the 2019 NSF SDR (four-year college or university, medical school, etc.), as the CGS categorizes institutions based on type of institution (e.g., research university, master’s/regional, liberal arts college, community college). Additionally, although the CGS’s classification of work activities is based on the NSF SDR, the 2019 NSF SDR has twice the number of options as CGS. These CGS revisions were made based on the experiences of practitioners using this classification system and their understanding of the shifting career landscape.

The survey asks for information on prior jobs, including secondary paid position(s), which can paint a fuller picture of past and current employment. For longitudinal data collection, the survey is administered to three alumni cohorts: those who are 3, 8, or 15 years past their PhD graduation, allowing for career outcome snapshots to be taken at different career stages. The 3-year cohort provides a window on recently graduated PhDs that supplements the NSF SED results; the 8-year cohort provides an opportunity for those who entered postdoc positions directly after PhD training to report on their career status; the 15-year cohort allows alumni to share mid-career experiences and any subsequent career changes. The survey has evolved since its inception to accommodate participant feedback. As a result, there are several versions of the Alumni and Student Surveys that require institutions to map or crosswalk the data in meaningful ways in order to present and interpret it. While the CGS and NSF surveys are similar, comparing results between them can cause challenges because they classify outcomes in different ways.

CGS published a series of research briefs^15^ based on their analysis of aggregated institutional data. The goal of these briefs is to help campus leaders and analysts contextualize institution-level data, especially in light of the national landscape of PhD career outcomes, while at the same time to continue a conversation about the skills and resources needed for student success in today’s PhD career landscape. Participating institutions choose how they want to share institutional data. For instance, the University of Wisconsin-Madison has a website dedicated to its participation in the CGS project with information about project goals, data briefs, project highlights, and project timeline.^16^ A majority of data visualizations are created using Tableau or other common data tools that institutions have licensure with, as well as the simple charts enabled by Excel exports.

### 2.4 First-destination survey, national association of colleges and employers

The National Association of Colleges and Employers (NACE)^17,18^ aims to provide thought leadership on the relevant issues and trends affecting the college-educated workforce; in doing so, they established national standards and protocols to guide higher education institutions in collecting and disseminating graduate outcomes data. Reporting categories broadly fall into the following: employment status (e.g., employed full time, employed part time, volunteer, seeking employment, seeking further education, etc.); mean and median salaries (full-time employed only); and bonus mean and median. Schools are encouraged to collect other information such as job title, employing organization, and position location, but it is optional to collect this information and these data are not reported to NACE.

NACE has collected first destination data on undergraduates for many years. In 2012, they established national standards for NACE member institutions to collect undergraduates’ first-destination outcomes. In 2015, NACE released another set of standards and protocols for collecting information from graduate populations, including both master’s and PhD programs.^19^

A key benefit of this taxonomy is that it gives NACE-member institutions that were not already collecting graduate program outcomes data a structure to report data. This structure aligns with surveys that were already being used for undergraduate outcomes, thus allowing institutions already collecting outcomes of undergraduates to avoid major changes to their survey by applying a similar methodology in order to collect graduate career outcomes. The NACE methodology also encourages reporting “knowledge rates,” e.g., reporting the relative percentage of graduates for which an institution has reasonably verifiable information about their outcomes—whether, for example, from self-reported information via surveys, information obtained through public searches (e.g., LinkedIn), or the employers themselves. A limitation of this taxonomy is that there is no industry associated with employers, and job titles are self-reported and not standardized with definitions. Job titles are not reported to NACE, though individual schools may report these on their websites. The University of Pennsylvania Career Services reports are an example.^20^

NACE reports outcomes both through written reports^21^ and through an interactive Microsoft Power BI dashboard^17^ that displays graduate outcomes approximately 6 months after obtaining their degree. The report can be viewed and/or filtered in many ways, including by degree type (B.S. or M.S.), institution type (i.e., private or public), Carnegie classification type, country, region, Classification of Instructional Program (CIP) code, etc. Prominent within the visualization are salaries and bonuses by career outcome. The outcomes for doctoral degrees are not included in the interactive dashboard but are included in the written report. Furthermore, the report provides the “knowledge rate” mentioned above, as well as the relative percentage of graduates with a known career outcome. The report displays the percent of employed graduates, those that continued their education, individuals seeking employment, graduates who entered the military, and individuals participating in a post-graduate fellowship or internship.

## 3 Systems for classifying career outcomes: institution or consortium-developed systems

Institution or consortium-developed systems have the benefit of being more agile to change with workforce development needs and may be more closely tailored or customized for specific fields or subfields. However, they may be less frequently used and hence the data may be harder to compare across fields. Solutions to address this problem are proposed in the discussion, including the crosswalk tool developed and presented herein. These solutions can result in compelling data to present to governing agencies (e.g., funding requests for program development or renewal). For example, data compiled across taxonomies could be used to advocate for legislative bodies, such as Congress in the U.S. context, to draft legislation for establishing sustainable funding mechanisms to develop and expand access to and utilization of career outcomes data. Proliferation of robust career outcomes data to inform practice and policy would be invaluable for creating change in the scientific enterprise including at the institution-level, leveraging the impact of federal evidence-based policymaking (Malloy et al., 2021).

### 3.1 Unified Career Outcomes Taxonomy

In collaboration with RBR, the NIH-BEST Consortium’s doctoral outcomes data was combined with categories used by the Office of Career and Professional Development at the University of California, San Francisco, to yield the three-tiered Unified Career Outcomes Taxonomy.^22^ The UCOT has three classification tiers: Sector: broad area of the workforce in which an individual is employed (e.g., academia, government, for-profit, nonprofit, and other); Career Type: broad type of work performed by an individual within their sector of the workforce (primarily research, primarily teaching, science/discipline-related, not related to science/discipline, further training, and unknown); and Job Function: identification of specific skill sets and/or credentials required for employment within that career type (e.g., “science writing and communication,” “science education and outreach,” “science policy and government affairs”). This tiered approach allows grouping, while also making distinct the function of each role.

The NIH-BEST consortium curated doctoral outcomes data among Consortium institutions, with the goal of cross-institutional assessment of evidence-based, promising practices for the career development of biomedical PhDs. However, it became clear that the data could not be compared, because each institution curated the data using a variety of different interpretations of the same terms. In an effort to create consistency and reliability in career outcomes reporting, the NIH-BEST Consortium member institutions formed a working group to develop a taxonomy for use within the Consortium (Mathur et al., 2018a). The UCOT provided an initial set of standardized definitions to common terms, which were later empirically tested and clarified to address identified areas of uncertainty. The taxonomy was iteratively tested by Stayart et al. (2020) to determine the classification consistency across different “raters”; this work resulted in a supplemental guidance document on how to interpret various cases, such that definitions would be applied consistently by practitioners who were curating the data. The results of this empirical validation process (Stayart et al., 2020) suggested that reliability improved with all tiers, and improvement occurred even when using non-experienced coders; this experimentally tested, updated version of the UCOT taxonomy was termed UCOT-Exp2.

UCOT is amenable to the addition of customized tracking “flags” for additional granularity that permits further interrogation of the data. This was particularly notable for categorizing faculty appointments, because the flag system permitted the identification of faculty rank and function (e.g., research, teaching, service) and simultaneously identified careers within academia and industry that could be grouped together by common job functions (e.g., leadership, strategy, internal policy, external relations, etc.). It has been rigorously and experimentally tested, with a detailed guidance document explaining how to categorize various positions. It can be adapted to track those in other disciplines beyond STEM. As an example, Wayne State University has adapted UCOT to the humanities by replacing “science-related” with “discipline-related.” The third tier of the taxonomy can be further adapted by adding additional job functions that are applicable to disciplines outside of STEM (Stayart et al., 2020; Mathur et al., 2018d).

Institutions utilizing UCOT, including institutional members of the CNGLS^3^, report their outcomes data with a wide variety of platforms and visualization methodologies, such as Tableau, static pie charts, and bar graphs. CNGLS members commit to reporting on at least the first two tiers of an earlier iteration of the UCOT taxonomy. A team at the University of California-San Francisco has published a detailed toolkit outlining how they track outcomes using all three tiers of the earlier UCOT iteration (Silva et al., 2019).

### 3.2 National Institutes of Health Taxonomy (NIEHS-based)

The National Institute of Environmental Health Sciences (NIEHS) developed a three-tiered, hierarchical taxonomy in which postdoctoral fellows are classified by “job sector,” “job type,” and “job specifics.” A detailed description of the taxonomy and how it was developed can be found in Xu et al. (2018) and in the alumni career outcomes dashboard^6^. First, Job Sectors describe the broad, overarching areas in which individuals are employed (e.g., academia, government, nonprofit organizations, for-profit organizations). Second, Job Types reflect the relative position levels in which individuals are employed (e.g., tenure-track positions, non-tenure-track positions, training positions, upper-level management positions, mid-level professional staff positions, supporting staff roles). Third, Job Specifics refer to the duties individuals specifically engage in through their respective positions (e.g., primarily basic research, primarily teaching, primarily applied research, science writing & communication, and regulatory affairs). A complete list of *job sectors*, *job types*, and *job specifics* can be found in the Supplemental Files compiled by Xu et al. (2018).

This taxonomy was developed using a “bottom-up” approach, meaning that the career outcomes of NIEHS postdoctoral fellows (who are primarily in the life sciences) were examined, and the designers considered how to best logically bin these career outcomes into categories. The outcomes of nearly 95% of alumni who left NIEHS between 2000 and 2014 were determined by extensive internet searching and validated by cross-checking with administrative data. Despite developing this with life sciences alumni, the taxonomy has universal applicability for classifying those in both the life sciences and humanities—especially the first two tiers (job sectors and job types). Many of the categories within the third tier (job specifics) are also universally applicable—adopters of this taxonomy could simply add additional categories within the job specifics section to fit their needs (e.g., primarily social science research).

The three categories (job sectors, job types, and job specifics) are independent of one another. It is unique in that it attempts to codify the relative position level (e.g., management versus support staff, etc.), which helps address questions about under-employment. It is broadly applicable to the sciences and humanities. A benefit to the “bottom-up” approach in developing this taxonomy is that the external labor market guided classification of careers. The system contains detailed definitions of each category, as well as example job titles. Additionally, a sample guide is provided that shows how to classify anonymized alumni working for a particular employer, with a given job title, doing a particular type of work activity. It does not capture fine detail regarding faculty-like positions (e.g., adjunct, tenured versus tenure-track). For example, all faculty-like positions are categorized either as tenure-track (which is all-encompassing of tenured, tenure-track, group leader, principal investigator, etc.) or non-tenure-track (research assistant professor, etc.). When using the taxonomy in practice, care should be taken when distinguishing whether to categorize a position as “professional staff ” or “management”–management-level classification is typically reserved for those in upper-level leadership positions, often serving in Director-type or Vice-President-type roles.

The NIEHS postdoctoral career outcomes data are visualized in a variety of ways in order to glean additional insights regarding career outcomes^6^. Briefly, the following were used (all based in the R platform): Directional chord diagram, Sankey, Bubble Matrix, Donut, Diverging bar chart, and Geographic visualizations.

### 3.3 Track Report and Connect Exchange (Canada)

Track Report and Connect Exchange (TRaCE)^23^ is a project headquartered at McGill University’s Graduate and Postdoctoral Studies group that aims to track and report on career pathways of PhD graduates, and serves any Canadian institution that would like to partner with them in tracking alumni. The current project tracks humanities, social sciences, and fine arts graduates using both quantitative and qualitative measures, with data collection through surveys, data scraping and more recently, narrative interviews. Data collected by surveys and data scraping is used to quantitatively assess overall career outcomes; data collected by narrative interview is reported separately to showcase the stories of how individual alumni navigated their careers.^24^ Career outcomes information collected by survey and data scraping for quantitative analysis includes three main categories. The first category is Employment Sector (e.g., academic, government, for-profit, non-profit, etc.), followed by Main Field of Employer (e.g., education, public and human services, STEM-related, etc.), rounded out with Job Function (e.g., academic research/teaching tenure status, administration, etc.).

The current iteration builds on two prior projects: a one-year pilot study in 2015–2016 that tracked humanities graduates; and the TraCE 2.0 project in 2017–2019 that tracked graduates in the humanities, social sciences, and fine arts.

Researchers adhered to a strictly standardized protocol when classifying higher-level data (e.g., sector). When categorizing more granular information, such as job functions, the categorization was variable. The research team acknowledged difficulty in categorizing faculty positions and chose to categorize them as non-tenure-track by default if a position’s tenure status could not be verified. While this step may avoid overestimating the number of individuals in a tenure-track-type position, it may have the unintended consequence of underreporting the number of individuals entering tenure-track positions. The demographic information collected in the surveys extended beyond basic information and included detailed options for one to self-report their gender identity and sexual orientation.

Data and narratives from 2008 to 2018 graduating cohorts at McGill University are visualized via an executive summary.^25^ Additionally, a quantitative report disaggregates the McGill data in many ways, including a detailed breakdown of the self-identified sexual orientation of participants^25^.

### 3.4 University of British Columbia Career Outcome Survey (Canada)

The purpose of the UBC Career Outcome Survey^26^ was to systematically determine the career outcomes of its doctoral students who graduated with a PhD between 2005 and 2013. The UBC taxonomy has two main categories, Employment Sector and Job Titles. Employment Sector includes Higher education, Not-for-profit, Private sector, Public sector. In addition, Higher education professionals are further subclassified (e.g., research-intensive faculty, teaching-intensive faculty, postdoc, administrator, term faculty, associate researcher). Job Titles includes a list of job titles that alumni currently hold. Further details, including a list of the definitions created for each field within the taxonomy, can be found on the UBC website.^27^

UBC’s Interdisciplinary Graduate Studies Program (IGSP) conducted a pilot survey in 2015 to assess the career outcomes of their graduates. In 2016, UBC extended the project to all of their PhD alumni as described above. The survey was designed to minimize the time required to complete it in order to maximize the number of individuals who would take it. Information on those receiving PhDs in philosophy and English were collected through the national TRaCE project, which, as described previously, collects information on humanities PhDs for partnering Canadian institutions. The authors used a multi-pronged approach, wherein both surveys and analysis of publicly available data were used to categorize outcomes, of which information was obtained for 91% of graduates. Approximately half the students responded to surveys, and thus information on the remaining students was obtained through internet searches. Survey responses were double-checked according to the alumni’s position and employer, and alumni miscategorizations (relative to UBC’s established taxonomy) were corrected.

UBC compares career outcomes data across various disciplinary groupings by also classifying programs according to the Statistics Canada Classification of Instructional Programs 2000^28^, the categorization system used for sharing U15 university data. This has the advantage of allowing comparisons of outcomes by discipline, rather than only at the individual program level, which is advantageous due to increased group sizes. Another strength of this study is that the authors make an effort to comprehensively research each and every position when they are not clear, so as to best characterize it rather than relying solely on either a survey response (which can be miscategorized), or by observing employers and job titles only at a surface level. With the current taxonomy, while more detail is available on what individuals are doing within the higher education sector, details are limited on the type or level of work being carried out in other sectors.

The PhD Career outcomes are publicly available online via an interactive dashboard using common visualization types (bar graphs, pie charts, etc.). The data are disaggregated in several ways, including geographic movement, job location, employers, job titles, gender, domestic versus international, and even down to data source (survey versus internet search). Career outcomes can also be visualized by sector of graduating discipline as well as the specific program of study. A comprehensive report that provides additional visualizations as well as alumni profiles was created and disseminated.

### 3.5 University of Toronto-10,000 PhDs Project (Canada)

The purpose of the 10,000 PhDs Project at the University of Toronto (U of T)^29^ was to determine the current (2016) employment positions of 10,886 individuals who graduated from U of T from 2000 to 2015 in all disciplines. Career outcomes were categorized into two major categories, Employment Sectors (e.g., post-secondary education, private sector, public sector, charitable sector, etc.) and Job Functions. Within each Job Sector, relative position types or job functions were assigned (e.g., within the post-secondary education sector: tenure-track professors, full-time teaching stream professors, etc.; within the private sector, based on the Government of Canada’s employment categories: arts, trades, biotechnology, finance, etc.). A list of definitions and a detailed guideline for coding are available in supporting material prepared by Reithmeier et al. (2019).

The employment positions were obtained by performing internet searches of publicly available sources such as university, government, company and personal websites, and directories and individual LinkedIn profiles, with ~85% capture success. The School of Graduate Studies (SGS) provided the names of graduates by year and their respective graduate unit/department, gender, immigration status, supervisor and thesis title. No individuals were contacted during the course of this project. Alumni survey instruments were considered, but previous studies indicated low returns and the potential for bias based on small sample sizes (Jonker, 2016). Some departments connected with their alumni to create compelling career narratives for their websites.

A strength of this classification system is that it contains granular data on the career outcomes of PhD graduates beyond broad generalizations. These researchers provide a detailed framework that describes each category’s definition—along with an in-depth rationale and logic framework underlying the decision process for classifying individuals in a certain manner (Reithmeier et al., 2019), and describe the painstaking lengths to which they went to identify and verify alumni through public sources—listing their commonly used internet sources that provided the most reliable data on alumni career outcomes. The U of T researchers also describe how faculty title designations may differ across international barriers, and they provide recommendations for ascertaining the relative equivalencies between Canadian (and U.S.) faculty titles and those from international (non-U.S.) universities.

The PhD career outcome data is publicly available on the SGS website using an interactive dashboard^29^. The data can be searched by division, discipline, gender, and immigration status. A 10,000 PhDs Project Overview and Divisional Fact Sheets with clear infographics, created using Tableau, can be downloaded. The 10,000 PhDs Project was initiated as a research project using student researchers with a peer-reviewed publication in PLOS-One as one of the desired outcomes (Reithmeier et al., 2019). The U of T has also joined the Coalition for Next Generation Life Science (CNGLS), and they have updated their interactive dashboard to report their career outcomes according to the standards set for those joining CNGLS, which includes reporting via the UCOT 2017 (Mathur et al., 2018a) taxonomy format. Reporting data based on these two different taxonomies allows readers to see how similar the Canadian employment sectors are relative to those within the 2017 UCOT, and further updated (UCOT Exp2) based on experimental evidence in Stayart et al. (2020).

## 4 Systems for classifying career outcomes: professional association-developed systems

Professional associations with broad disciplinary coverage (e.g., American Association of Universities Data Exchange) may develop classification systems with a broad variety of options in addition to intentionally incorporating standard measures – all while protecting their flexibility to expand upon prior definitions. In contrast, professional associations that are based in field-specific societies (similar to institution- or consortium-developed systems), may produce classification systems that, while likely to be nimble and able to change with workforce needs, may have narrower yet more customized and fleshed-out career options commonly associated with that field. This high level of detail could be valuable when members of the society or association – whether early career members, senior researchers, or individuals – advocate to lawmakers for support that would help sustain research projects. However, field-specific systems may not provide representation of uncommon job areas for that field, even if trends are changing. This is certainly the case for roles in policymaking, for example, where in some cases, particular scientific expertise is valuable to specific scientific societies, but more often than not, executive and U.S. Congressional advisory roles rely on knowledge of scientific practices and the use of evidence-based practices in policymaking regardless of discipline (Oliver et al., 2014).

### 4.1 American Association of Universities Data Exchange

The American Association of Universities Data Exchange (AAUDE) taxonomy^30^ effort takes advantage of existing government employment classification methodologies, including the jointly developed U.S., Canadian, and Mexican North American Industry Classification System^31^ (NAICS; the coding scheme for industry) and the U.S. Standard Occupational Classification system (SOC; the coding scheme for occupation, US Bureau of Labor Statistics from 2018)^12^. Thus, the AAUDE taxonomy relies on a well-understood, user-friendly coding system where jobs are classified by industry (sector) and occupation (or function). Both industry and occupation have a primary (“major”) and a secondary (“minor”) level, thus enabling fairly specific classifications without being overly detailed and thus burdensome for coders. It also does not require extra “tags” or other designators to code level of work (such as managerial-level or tenure track). There are four mandatory fields used to classify alumni in the AAUDE taxonomy. The first category is Top-Level Employer (Industry) Type (e.g., Academic, Industry, Non-profit, Government, Entrepreneurial, Freelance). This is followed by the Second-Level Employer (Industry) Type (e.g., Institution type or NAICS industry code). Third, the Top-Level Occupation (e.g., further study, academic career stage, other research position, other full-time work, exclude from cohort, other, includes non-work occupations such as travel) is reported, followed by the Second-Level Occupation (e.g., academic career stage, SOC (occupation code), “other” detail). A full description of the taxonomy was published online.^31^

The AAUDE system, although based on both NAICS and the SOC, sometimes combines or sometimes excludes certain categories or levels of classification, and adds its own categories for academic careers. This classification system was designed to improve data-sharing about PhD career outcomes among AAU institutions: to enable cross-institutional comparisons, a working taxonomy was created in 2017, and then refined in 2018, resulting in the version (Version 6) described above.

A strength of this classification system is that it is sufficiently detailed to capture nearly all career outcomes of PhD alumni across disciplines but is not so detailed that it would take a coder extensive time to code an employment outcome. It is able to reach broadly across disciplines and career pathways, and is thus potentially more useful in coding employment outcomes for humanities students than other existing coding systems. AAU institutions are required to provide their outcomes to AAU annually using this taxonomy. The NAICS and SOC codes are officially used by government reporting agencies, providing robustness and longevity (these codes are available publicly and can also be utilized by trainees for career exploration in O*NET^32^ – the career portal using the federal workforce classification system). As a result of the AAUDE taxonomy’s wide use, the data can be compared across institutions and over time. Furthermore, this coding scheme is one of the three being used by the consulting firm Academic Analytics^33^, which has been adopted by some universities to gather career outcomes data. A limitation of the underlying SOC and NAICS coding schemes is that they are updated infrequently, such that newer career paths may not be represented (created in 1977; last updated in 2018 but revisions can take up to 10 years)^12^.

A large number of institutions collect information as part of the AAUDE initiative. In one such example, Texas A&M University reports the results from their AAUDE Doctoral Exit Survey^34^, which includes interactive drill-down options, enabling one to visualize differences in career outcomes from a variety of groups, including those from different ethnicities.

### 4.2 American Historical Association

The American Historical Association (AHA)^5^ serves historians in all professions. As part of serving its constituents, AHA embarked on a project to identify the career outcomes of historians on a national scale. In doing so, it developed a taxonomy to classify their careers. Similar to the AAU taxonomy described above, the AHA taxonomy also includes standard SOC codes from the Bureau of Labor Statistics. The taxonomy collects information in two main categories. First, Sector (e.g., government, academia, for/non-profit, etc.)–which notably also includes further academic granularity such as definitions for higher ed. admin/staff, post-doc, and variants of 2- or 4-year tenure- or non-tenure-track positions; not-found and retired/unemployed are also included in this category. Second, Job Function is based on SOC codes^12^ from governmental standardized definitions for job functions.

Among the vanguard of PhD career outcome transparency, the “Where Historians Work” AHA taxonomy was developed as part of an initiative funded by the Andrew W. Mellon Foundation to track career outcomes for historians nationally and was published as a summary report (Swafford and Ruediger, 2018) and interactive dashboard^5^.

This approach is comprehensive, including all historians graduating with PhDs nationally between 2004 and 2013. This list of historians was ascertained by analyzing the names and dissertation titles from the AHA’s Directory of History Dissertations. Using these data, AHA searched publicly available online sources to determine the career outcomes and found data for 93% of historians using the AHA’s Directory of History Dissertations. Institutional and personal data (e.g., specialization area, PhD department, geographic area, and gender) were collected, analyzed, and connected with career outcomes data (Swafford and Ruediger, 2018). Since this project was intended to serve the needs of humanities (historians specifically), it may not capture some common social sciences or STEM career outcomes (e.g., a category cited as common for historians such as “Library/Museum/Archive” may be less applicable for scientists). By using SOC codes, the AHA taxonomy is versatile and can be benchmarked alongside other groups using these same governmental standards. It includes less common job functions that may be applied more broadly by disciplines outside of history.

The results of this study are available to explore on an interactive Tableau dashboard^5^, and include a variety of visualizations, such as tables, geographic locations, and packed bubbles. Data can be filtered down to reveal the name of the PhD-degree granting department, specialization, cohort, or gender, depending on the specific visualization at hand. Swafford and Ruediger (2018) provide a concise overview of the different stories that can be told by examining AHA’s dashboard.

### 4.3 Modern Language Association

The Modern Language Association (MLA)^35^ is the professional association for English and Foreign Languages which also embarked on a project to identify the careers of Modern Language PhDs. Similar to AHA, MLA also received a grant from the Andrew W. Mellon Foundation to collect information relating to PhD graduates. To collect this information, MLA surveyed a random sample of PhDs. In the survey, respondents were asked to report where they were first employed, and two tiers of outcomes were collected. First, Job role (e.g., tenured faculty, tenure-track faculty, administrative, employed outside higher ed., etc.) and second, Job Specifics (varied based on job role: e.g., full time, postdoc fellowship, business, government, non-profit, etc.). In addition to questions relating to their career path since graduating, respondents were asked questions about their job satisfaction and earnings. A full description of the methods and findings was published online.

The impetus for the survey arose from concerns relating to the shrinking number of full-time tenure-track positions advertised at postsecondary institutions, as well as a desire to learn about the full range of careers pursued by PhD graduates. The report generated from the findings of the 2012 MLA Survey was published in 2015 as “Where Are They Now”.^36^ In 2017, the MLA contacted individuals from the earlier 2012 survey and invited them to complete a new survey about their employment since they first received their doctorate.

This survey aimed to measure the career progress of responders as opposed to only looking at first-destinations post-PhD. It permits a discrete time-based understanding of the career outcome landscape of PhD graduates of English and foreign languages and thus has application in curricular programming to better prepare doctoral students for a range of careers. Unlike the U.S. federal government’s SDR^11^, this study is not longitudinal. As survey respondents were anonymous, the 2017 survey responses could not be linked to the earlier 2012 survey responses; this prevents knowing exact movement from one career into another. Nonetheless, it allows one to observe trends/changes in career outcomes of the overall cohort from one time period to the next. These career outcomes should be interpreted with caution, as the size of the survey is small; of the 1,949 survey respondents for whom email addresses were found, only 310 responded to the survey.

For questions relating to type of employment, the report includes a data table as well as pie charts, columns, and cluster columns to render the data easy to read and clear. For all other questions, the report includes pie charts, columns, and cluster columns. All of the visualizations accompany text-based data analysis.

### 4.4 Association of Schools and Programs of Public Health first-destinations data collection

The Association of Schools and Programs of Public Health (ASPPH) is the membership association representing schools and programs of public health which are accredited by the Council on Education in Public Health (CEPH), and includes more than 111 schools and programs which provide bachelor’s, master’s and doctoral (PhD and DrPH) degrees in the public health disciplines. Beginning in 2014, ASPPH began collecting first-destinations employment outcomes data from member schools and programs, gathered 1 year post-graduation. Data is gathered by participating schools and programs and reported to ASPPH annually, including six categories. The first category is Employment Outcome (e.g., full-time employed, part-time employed, employed in a fellowship or residency program, continuing study); whereas the second category is Sector of Employment (e.g., government, academia, for-profit, non-profit, hospital/healthcare) and sub-sector (e.g., local government health department, local government not health department, pharmaceutical company, consulting firm). The third category establishes whether the position is new post-graduation, or a continuation of existing employment; whereas the fourth category includes subjects of further study, for those pursuing additional education. The fifth category reports salary and bonus and the sixth reports degree debt from public health degree. A full description of the methodology and initial findings was published in the American Journal of Public Health; a detailed description of the taxonomy, including definitions, is found within the Supplemental Appendix (Plepys et al., 2021).

The ASPPH data collection effort was initiated in response to broader needs for data on the public health workforce as well as the outcomes of public health graduates at the bachelor’s, master’s, and doctoral (PhD and DrPH) levels, which were not systematically or consistently captured in the past (Krasna et al., 2021b).

The taxonomy was originally designed in 2014 as a pilot project^37^ to increase enrollment in public health degree programs. It was designed to be used in tandem with reporting for the Council on Education in Public Health, the accrediting body for public health schools and programs, which requires schools/programs to report on employment outcomes but did not have a set standard for collecting data. The “common questions” used in the pilot were formulated with input from ASPPH member schools and programs; the data collection instrument was designed by the ASPPH Data Advisory Committee.

The final collection includes data from a total of 64,592 public health graduates from four graduating cohort years from 2015 to 2018, of whom 53,463 had known outcomes. Data was gathered each year, and the number of schools and programs reporting to ASPPH increased from 55 institutions in 2015 to 111 institutions in 2018.

A feature of note is the detail collected within the sub-sectors, which includes those that are particularly relevant to public health. For example, finer detail on government employment (e.g., “state health department,” “state government, not health department,” “local (county or city) health department,” “local (county or city) government not health department,” “tribal government”), and the for-profit sector (e.g., “pharmaceuticals, biotech, or medical device firm,” “health insurance company”) are uniquely positioned to capture details on the public health workforce. Because doctoral graduates in public health are often hired by government agencies, pharmaceutical firms, consulting firms, and so on, this level of detail provides further insight into the connection between these graduates and the public health and healthcare workforce beyond academia. Second, another key feature of this taxonomy is that “Healthcare” is one of the major categorical sectors, rendering it unique (along with TRaCE) among the taxonomies described herein. This could be especially beneficial for identifying public health graduates within the healthcare field. However, it would not be as straightforward to compare across other taxonomies who parse healthcare as a subdivision of other major sectors (e.g., University-associated hospitals may be categorized as Academia in other taxonomies; government-associated hospitals, e.g., Veteran’s Affairs, may be categorized as the Governmental sector in other taxonomies). Additionally, the survey also gathers data on student loan debt, salary and bonus. As this taxonomy was developed to look at discipline-specific career outcomes of those with a public health degree, it serves as an example of the benefits gained by using a specific taxonomy for specific constituents.

The publication citing these data primarily reports outcomes by degree level and area of study using tables; the results were analyzed primarily with descriptive statistics, with employment outcome status being compared by area of study.

## 5 Career outcomes: data visualization

In addition to identifying career outcomes and classifying them according to a taxonomy, it is important to communicate these data in an effective manner including to the federal government. In the age of big data, a wide range of visualization methodologies and platforms have become available that can be leveraged for identifying and sharing career outcome trends. Different visualization techniques can be used depending on the intended purpose and audience. For example, answering different questions about whether there is a need to depict how students from different programs have significantly different outcomes, how career outcomes have changed over time, how individuals have migrated from training locations to employment locations, or how individuals from underrepresented backgrounds have career outcomes that fare differently than those from well-represented backgrounds, can help determine the choice of how to visualize the data. These data could be especially useful for federal government staffers and decision-makers to utilize when setting policy priorities on ways to support the future research workforce based on where PhD graduates are employed and who is/is not currently represented (Benderly, 2018). Thus, thoughtfully crafted graphs and charts that tell a clear story could be used by government staffers when drafting legislation to support the future of the PhD workforce; career development professionals and other university administrators could serve as expert resources for these staffers in their work when it comes to higher education data. In best showcasing this data clearly, several resources are available to help inform the decision-making process around which visualization method to use.^38,39,40,41^

### 5.1 Visualization scope

Visualizations were selected in part by reviewing the Graduate Career Consortium Outcomes Database (Collins et al., 2020) and showcasing examples that depict multi-dimensional outcomes in innovative, meaningful ways. Resources, tools, and coalitions were selected for inclusion by reviewing published reports (including conference abstracts), literature, and websites from within the past 10 years—with information more commonly being found from within the past five. All of the referenced websites contained within this manuscript link to beneficial resources, and it must be noted that website addresses are prone to change. The links shown herein are current as of 2023. Supplementary material provides both examples and a comprehensive synthesis of methods for visualizing PhD career outcomes (see Supplementary material 1: Visualization Platforms and Types), along with tools, resources, or organizations that assist constituents in collecting and reporting this information (see Supplementary material 2: Resources, Tools, and Coalitions).

Apart from telling a story, it is also worth considering the way data are visualized so that the data can be comparable for benchmarking purposes and to reduce the likelihood of misinterpretation. As an example, consider whether or not the “unknowns” are included within the dataset being visualized. If they are excluded, then the career outcome values are artificially inflated relative to the true population, since the denominator is artificially smaller by excluding unknowns. As another example, if one were to visualize a subpopulation within an overall student alumni cohort and represent that subpopulation on a scale of 0–100%, a casual reader could easily misinterpret this as representative of the total population, especially if the data were shown out of context. Thus, care should be taken to ensure that visualized data are clearly labeled in all cases. As a way to assess labeling clarity, assume the figure or visualization of the data will stand alone—consider if taken out of context in this manner, whether the data could be easily misinterpreted. If the answer is “yes,” then the author should either label the figure more clearly or represent the data in a different manner altogether. This is increasingly relevant in the age of social media when snippets, excerpts, or visualizations are commonly highlighted out of context.

## 6 Discussion

The call for transparent PhD career outcomes reporting has led to the rapid development of taxonomies from a variety of sources, including national classification systems, institutionally developed systems, and professional society systems. Each of these approaches has benefits, such as either more broad or more specific applicability, the inclusion of either more nuanced or less nuanced field-specific career categories, and the availability of either highly accessible classification options (e.g., with replicable instructions widely/freely available) or those less accessible (e.g., due to an associated fee, but providing the ability to use a novel classification system quickly). Yet no matter which classification system is selected, if it is not displayed and comprehensible via effective data visualization, arguably it may not truly be transparent. Implications of transparent data collection, classification, and dissemination can have wide-ranging impacts on economic development, training program design, and educational curriculum development, among key outcomes of interest.

Clear taxonomic classifications for PhD career outcomes can prove to be valuable resources for policymakers who can utilize this information in encouraging agencies to tailor their offerings to the needs of this population. One-pagers with clear and consistent career outcomes data across universities would also be valuable for advocating to government staffers on the need for increased funding of existing PhD programs or the development of new programs. Finally, in the case of STEM PhDs, these data can be useful for new legislation being drafted by Science Committees and their staff to support the STEM pipeline. These outcomes data can be used by associations and coalitions of universities/varied institutional types that represent the higher education community as strong evidence underlying recommendations to those in government who have the power to take action (Overseas Development Institute, 2017). It is necessary for universities and associations to develop recommendations for change based on the data presented in the career outcomes; some examples include the need for better links between training and job prospects, supporting existing programs, or creating new programs to bridge the gap between research, education, and labor. The importance of a unifying taxonomy includes being a useful resource for national to local governments to consider existing gaps and draft relevant legislation to fill them. Additionally, the unifying taxonomy could aid in crafting plans to develop executive branch programs that consider where national investments should be directed either toward funding more programs that enable data collection on PhD career outcomes, or programs that facilitate using the existing data to link training with job market needs for PhD graduates.

Additionally, many of the career outcome taxonomies described herein have broad applicability in international contexts. Core categories, such as employment sector and job function remain relevant across different national systems, even as specific job titles and career pathways may vary. For example, the taxonomy developed to track NIEHS postdoctoral outcomes (Xu et al., 2018) categorized outcomes from those who trained at NIEHS but became employed internationally (nearly 1/3 of the sample; see Supplementary material 1: Visualization Platforms and Types – Figure 3 – in which a sample Directional Chord diagram displays international employment migration trends). International job titles and functions are thus defined within the NIEHS taxonomy itself, and commonalities/differences between domestic versus international job outcomes are explained and mapped. By leveraging such frameworks and refining based on regional differences, international career outcomes can be more effectively analyzed, enabling policymakers and institutions worldwide to make data-driven decisions about training and workforce development.

### 6.1 Taxonomy/classification system commonalities and differences

As evidenced by the taxonomies described, a wide variety of methods for classifying the career outcomes of doctoral-degree holders exists. High-level characteristics of these taxonomies include unique developments and applications such as experimental testing and including narratives and skills. First, it is rare to find a taxonomy with experimentally tested reliability and validity (UCOT Exp2). Second, it is notable that one taxonomy combined data with narratives (TRaCE), with the added benefit of being offered comprehensively as part of a national project. Third, another project takes the approach of better understanding career outcomes by identifying the skills and professional development competencies tied to them (CGS PhD Pathways).

In addition to the unique qualities described above, some taxonomies were designed to capture career outcomes in specific fields such as public health (ASPPH), humanities (TRaCE, AHA, & MLA taxonomies), and STEM (such as the NSF SDR); whereas others were designed to be implemented across disciplines (AAUDE, UBC, U of T 10,000 PhDs, NACE, and NSF SED). In addition, however, some that were originally developed for STEM fields have been adapted or modified in fields other than their original disciplines (for instance, UCOT and NIEHS were developed for biomedical careers but can be applied across fields) – and yet, a strength of using a discipline-based taxonomy is that it may capture niche careers in that discipline particularly well.

Another commonality across some taxonomies is the reliance upon standardized common labor metrics (NSF SDR and AHA use the Bureau of Labor Statistics SOCs; similarly, the AAUDE relies upon SOC and NIACS). A benefit of taxonomies based in economic standard measures is that they can be more widely applicable and can be compared with other government data such as economic indicators, allowing comparison of career outcomes at institutions to national, regional, and local economic and market trends. Because these standardized classification codes have been developed and vetted carefully, they are widely representative of skills and career areas; however, a potential downside is that these may not be as updated as frequently (e.g., 10-year cycles), and thus they may not always reflect new or emerging fields. In addition, a limitation of the standardized codes is that, while expansive, they may not always be specific enough to accurately capture the variety of career outcomes specific to doctoral alumni. In contrast to using these common labor standards, the NIEHS taxonomy built their system off of the present labor market by first identifying outcomes and employers and then determining how to bin the outcomes into logical categories. The benefit of this approach is that it allows one to flex to a rapidly shifting career landscape, but the outputs cannot be compared to commonly used, robust standards.

Aside from comparing across the taxonomies themselves, one could also consider that the timing of data collected with these systems varies, including some collections which take place prior to, or near, graduation (SED), and those occurring approximately 6 months past graduation (NACE), 1 year after graduation (ASPPH), or at longitudinal intervals (SDR). Since it can take time for new graduates to find employment, data gathered shortly after graduation is likely to appear less favorable than that gathered a year or later post-graduation. Additionally, many institutions that are currently collecting outcomes data may capture single snapshots in time, such that the career outcomes they report are reflective of those who left within the past two decades. However one chooses to report, the data should be clearly marked so that individuals examining the data can understand the time in which a person graduated or left the institution (in the case of postdocs), and the time in which the career outcomes were collected.

Regardless of the system an institution chooses to classify the career outcomes of their alumni, we feel that it is imperative to identify which taxonomy was used, with clear references to the documentation of the taxonomy. This is crucial for individuals examining the data to have an accurate understanding of what the data mean and to ascertain the degree to which data from different departments or institutions can be compared. One suggestion for how to clearly delineate this would be to develop a universal shorthand methodology for tagging all reports and graphics with the taxonomy used. For example, consider the UCOT taxonomy—when the taxonomy was revised based on feedback and tests to ensure inter-rater reliability, a new version was published (Stayart et al., 2020). The original version is referred to as UCOT 2017, and the updated version UCOT-Experimental (UCOT-Exp2). This helps to clearly distinguish the specific taxonomy and version being used, which is important, given that taxonomy development should be viewed as a continuing process as career paths shrink and grow throughout our ever-changing economy.

To complement the description of the taxonomies described above, we collated and aligned the major taxonomic categories from a number of additional Universities and classification systems, including all of the taxonomies described herein to create a crosswalk tool (Collins T. R. et al., 2021; Collins T. et al., 2021). From these alignments, clear patterns and commonalities across classification systems are apparent, resulting in the emergence of an overarching primary list of terms and definitions. For example, all systems include: (a) employment sector in some form, and most also include, (b) position/job or career type, and/or (c) function/role/work activity.

Within the employment sector, eight clear categories appeared most frequently, including variations on the following (number of times sector included): Academic (30X), Government (30X), For-Profit (26X), Non-Profit (28X), Individual (27X), Unknown (21X), Not in workforce (23X), and Unemployed/Seeking (11X). Healthcare was represented as a standalone subsector in two taxonomies, but most taxonomies included healthcare systems within the other 8 sectors described (e.g., University-affiliated hospitals would fall under the academic sector while Veterans’ Affairs hospitals would fall under government, etc.). The main area of discrepancy in the sectors described included whether an entity was public or private, and how that was categorized across different taxonomies. For example, Universities could fall under either the public or private sector, as could primary/secondary schools. However, some taxonomies categorized only those at Universities within the academic sector, whereas those in primary or secondary schools were categorized according to whether they were either public/government or private/non-profit. Other taxonomies, on the other hand, categorized all educational institutions (whether preschool through universities) within the academic sector.

For the remaining two themes (position/job or career type and function/role/work activity), classification across the taxonomies was less consistent, but broad commonalities could be ascertained. Within position or career types, Faculty-like positions (encompassing those including tenured, tenure-track, tenure-unclear, and group/team leader) were the single most commonly observed category, appearing 45 times in some form. Other commonly observed categories were that of Non-tenure track (27X), Mid-level Professionals (13X), Senior Management (12X), and Trainee (35X). Less common, but appearing multiple times, were Unknown (3X), Discipline-related (6X), Non-discipline-related (3X), and Support staff (3X).

For job functions/roles/work activities, 31 categories were able to be aligned among the taxonomies examined, with some appearing at a much higher frequency than others. The full listing can be found in the crosswalk tool (Collins T. R. et al., 2021; Collins T. et al., 2021). Some of the most commonly appearing categories include those conducting research or teaching. These are often further subdivided in order to better ascertain the type of work being conducted (e.g., basic or applied research; full-time faculty teaching or science education/outreach, etc.). Categories most difficult to align concerned those within product development and manufacturing/engineering, as the design and development of products may involve engineering principles. Entrepreneurship was also difficult to align because it is sometimes considered a job function, while other taxonomies include this within the “sector” fields as the “Individual” sector.

The goal of collating and describing the taxonomic classifications for doctoral-level career outcomes is to assist institutions in determining the taxonomy that works best for their needs by highlighting key features, benefits, and caveats to different systems. There is little cost to choosing a taxonomy, but there is a high cost to *not* collecting outcomes data. This cost of not collecting outcomes may become evident in many ways—by obfuscating where students or postdocs enter into careers; in delayed curricular innovations; in difficulties during recruitment by not being able to speak on graduates’ outcomes; and in the cost of an institution not knowing how their doctoral graduates and postdoctoral scholars are contributing to innovations within the global economy and society as a whole. In short, there are many valuable taxonomies from which to choose—the truly costly choice would be to not select any system of reporting the graduate outcomes.

### 6.2 Importance of career outcomes data visualization

Career outcomes hold the most value when they are readily available and easily accessible. Data visualization is key to communicating PhD outcomes in a transparent manner (see Supplementary material 1: Visualization Platforms and Types) in order to connect trends in the data with policymakers who can utilize these data for future policy development. This is important for prospective and current members of the research workforce, including students, young researchers and potential recruits into future academic/research roles for whom these data may allow individuals to make informed decisions about the appropriate levels of education and training to support their career progression in research (degree choices, postdoctoral training, etc.). This is also important for prospective or current PhD students who need to see themselves in decision-making roles in research and policy, for which role models are important and these data can showcase individuals from different cultures and backgrounds who are part of the research workforce. Data visualization also supports federal policy-making at the highest levels in the Executive or Legislative Branch and state-level efforts needed to recruit and retain talent into the research workforce based on knowledge of the gaps that need to be filled and data on where graduates have gone (Mikell, 2023). Without information to evaluate and illustrate the importance of the value of advanced PhD training, it is impossible to advocate for research, education, and training using empirical arguments. Training environment decision-making (postdoc pay, graduate student numbers and their backgrounds, etc.) may also be impacted by the ability to query large amounts of data across a diverse array of career areas and training levels among universities. Enabling this data collection can allow university administrators, program directors, and faculty advisors to better inform internal policies with data to support the programs developed and to advocate for federal funding to support these programs. In sum, the extensive proliferation of doctoral-level career taxonomies, transparent career outcome tracking, and effective data visualization could indelibly transform the research workforce of the future. The authors add their voices to the call, in line with many others (RBR, FOBGAPT, FoR, NIH BEST, CNGLS, etc.) that have continuously advocated for doctoral career outcomes transparency.

Transparency in doctoral career outcomes can also be useful for universities to determine who is currently missing from the workforce, and a standardization across outcomes tracking and data visualization can be extremely helpful for decision makers in government to continue funding current programs or design new programs to fill the needs of the scholarly community. Depending on the data, government actions could include expanding current programs to students from under-represented backgrounds, including more women in science who are still a minority, or allocating additional funds to specific types of institutions, so that the research workforce within universities reflects the diversity of the nation. When it comes to visualizing data to present to policymakers, one-pagers with clear “asks” and concise data are the most helpful way to have conversations about policy changes intended to impact the research enterprise more broadly.

### 6.3 Future directions

Research should continue to develop and examine evolving taxonomies for subfields. In addition, it may be beneficial for a cross-disciplinary, unified taxonomy developed in the future – this could benefit from national stakeholder engagement and being embedded in federal standardized reporting organizations (e.g., NACE undergraduate career outcomes reporting; NSF longitudinal career outcomes reporting; NIH or other federal funding agencies). A unified taxonomy would be helpful for policymakers to evaluate research and higher education community gaps that can be filled legislatively or in the executive branch, which could occur in conversation with policy decisionmakers and their staff to design new initiatives to address research workforce needs. Such a taxonomy might also utilize the International Classification of Occupations^42^, which seeks to provide a framework to enable international comparison of occupational data; additionally, it will be important to follow work from the European Science Foundation, who also calls for a common taxonomy.^43^ In addition, it is important to maintain the ability to regularly adapt and add new PhD career options, particularly in light of technological and commercial advances that can impact job types available as well as novel and evolving workforce needs. At the same time, new career trends/trajectories are constantly evolving – with a salient current example of the rise of AI which will undoubtedly transform the job market through creation and elimination of job tasks (Abdous and How, 2023). University administrators should be familiar with how AI might impact research and training practices and should invite experts in AI or policymakers working on AI issues to their university to shed light onto these topics – the workforce could expand through federal programs that support innovations in utilizing AI in higher education and research training for PhD students. These transformational changes to the career landscape cannot always be anticipated, but nonetheless must result in evolutions and adjustments to common career fields and job titles. In paradigm shifts (Kuhn, 1962), imagining the new reality before it happens is literally not possible – but once the paradigm shifts, a new awareness of possibilities opens up.

There continue to be gaps in the literature, as noted by Van Wart et al. (2023), who demonstrate the need for more evidence-based research broadly, including for informing policymaking. For instance, career taxonomies have in some cases been developed without follow-on information to assess the validity and reliability of the instruments/classification systems created in order to improve the replicability of future work. Although some reliability testing has been conducted (Stayart et al., 2020), additional reliability and validity tests for classifications of career outcomes will prove valuable additions for future work to establish consistent tools that can be used in future research and implementation. Furthermore, more empirical research is needed to assess meaningful differences by taxonomic categories for PhD career outcomes. Use of taxonomies to aid in predicting outcomes, such as career trajectory, salary, skill differences, etc. (Sinche et al., 2017) will enable researchers and policymakers to better understand the career pathways available to PhDs in order to determine how this information can be utilized more broadly.

Already-identified factors affecting career outcomes should also be further studied in order to clarify their impacts on career outcomes, which can impact the makeup of the workforce (Brown et al., 2023). Such factors requiring further investigation may include the impacts of gender differences, citizenship status, social identity, sexual orientation, etc. on outcomes; knowing more about how these factors impact career outcomes can highlight important gaps in the workforce as they relate to who is being excluded from research careers and what the federal government can do to assist. Further relating to exclusion, a recent study shows that approximately 80% of hired faculty in the U.S. come from only 20% of institutions; this should be further extended to clarify the effects of such exclusion on the broader workforce. The use of a well-defined taxonomy for classifying the aforementioned faculty roles would enhance understanding of such results (Wapman et al., 2022).

Aside from institutions themselves, the location of training has been shown to be an increasingly important influence on career outcomes (Xu et al., 2018; Feig et al., 2016), and future work should examine if some career trajectories are more impacted by the geographical location of training than others (e.g., biotech/pharma in hubs like Boston, MA, San Francisco, CA, and Research Triangle Park, NC). There may also be differential impacts on the research workforce where these hubs exist. Cost of living and the availability of jobs may also play an increasingly large role for who is able to pursue careers requiring graduate training (whether academic or otherwise) based on where they live, which should also be taken into account by policymakers using these data to make decisions. Fields-of-study have also been shown to influence career outcomes (Xu et al., 2018; Feig et al., 2016), and more research is needed to identify and compare outcome trends that differ by field. More granular detail about the impact of these and other factors on career outcomes can be used to create customized reports about specific topics or to address specific communities of interest. Having more robust information about the variety of factors that influence PhD career outcomes could help shape future policies and programs.

Another future direction of research includes the impact of how AI may eventually be able to efficiently classify career outcomes reliably and automatically; while this has great potential and future promise to expedite career outcomes tracking broadly (free tools, etc.), it has yet to be developed and tested fully. This could ultimately vastly increase accessibility, which may be especially important to institutions that are less well-resourced when it comes to federal funding as this could allow for career outcomes tracking with less person-power and funds needed to conduct/implement. However, this development must be approached with caution, as anecdotal issues with reliability of classification are rampant (e.g., so-called AI “hallucinations” of nonexistent data reported with confidence; the lack of replicable results due to AI variations in repeat queries; problematic answers that rely on human-designed prompts that may produce errors). More federal funding may be needed to invest into AI research in order to understand these processes and develop reliable mechanisms for analysis before deploying these mechanisms at a large scale. There are other considerations, such as privacy preferences or concerns of alumni, and hence more regulation may be needed once AI is involved. Future directions should also include the potentially powerful positive developments that AI could facilitate in research and higher education, while also considering risk mitigation and ethics. Ultimately, these data can be useful for automating the process of data analysis and tracking with the caveat that risks may still be involved until and while the methodology is being honed.

The increased collection of PhD career outcomes data informs federal program directors and institutions, aiding data-driven decisions on program offerings and policy-making. Initiatives like NIH BEST, CNGLS and FoR, alongside government-developed taxonomies such as NIEHS, are helping to shape acceptable standards and practices (see Supplementary material 2: Resources, Tools, and Coalitions for a summary). These programs not only influence national expectations but also nurture future policymakers. For instance, programs such as UC Irvine’s NIH-BEST trains PhDs for careers in policy and government, fostering thought leadership (Bankston et al., 2023). These thought leaders and policymakers envision and shape the future research landscape, aiming to break entrenched paradigms and create conducive conditions for the future research workforce to thrive and advance science.

## 7 Conclusion

Tracking, compiling, and publishing PhD career outcomes are crucial to the future of the academic research enterprise and higher education, and are part of growing appeals for systemic change (NIH, NIH-BEST, Council of Graduate Schools, Graduate Career Consortium, National Academies, Coalition for Next Generation Life Science, Rescuing Biomedical Research, etc.). Efforts to gather and share career outcomes data can help inform training and education initiatives related to the decline of available faculty jobs (Langin, 2020), economic and workforce needs, evolving postdoctoral policies, institutional accountability, and growing diversity in the career paths available to PhD recipients (Sauermann and Roach, 2012). The maintenance of career outcomes data is thus becoming an essential practice for institutions that issue PhDs, and standardization of such practices will have broad and powerful implications. First, availability of transparent career outcomes reporting will empower prospective students and postdoctoral scholars to make informed decisions. Second, at institutional levels, access to career outcomes data will enable effective intra-institutional planning and decision making, will facilitate inter-institutional comparisons and benchmarking, and will enable institutions to evaluate their ability to address equity, diversity, and inclusion [e.g., whether gender or race and ethnicity affect outcomes; whether an institution’s training environment and structure equitably support all students/postdocs as they navigate into careers (Mathur et al., 2018b,c; Porter et al., 2017; Rohde et al., 2020; Hart and McKinney, 2020; Baas et al., 2018), etc.]. Third, systematic accessibility to career outcomes data will allow for nuanced analyses and effective visualization (e.g., Murphy, 2013; Bhombe et al., 2019; Cuzzocrea and Mansmann, 2009), empowering policymakers to make informed decisions based on increasingly accessible evidence that can be used to identify important economic, labor, and biomedical workforce development trends (e.g., Forsythe et al., 2020; Krasna et al., 2021a; Russ et al., 2016; Krasna et al., 2020; Lenzi et al., 2020). As discussed, the standardization and use of common metrics to inform policy can provide clarity in comparative results; accordingly, there have been several efforts to create unified career taxonomies to better represent national, global, and cross-disciplinary trends [e.g., UCOT in the U.S. (Stayart et al., 2020), and the ISCO internationally^42^]. Hence, while many aspects of PhD career taxonomies are broadly applicable—emerging literature has also begun to explore differences in PhD career classifications, factors, and trends across countries, cultures, and continents (e.g., McAlpine et al., 2021; Neumann and Tan, 2011; Yang and Fumasoli, 2024; Wenqin et al., 2018). Future policymakers may benefit from balancing their reliance upon generalized standard metrics to gain a broadly applicable understanding of economic and career outcomes with more granular insights that may be gained by using tailored, novel, and evolving measures of career outcomes, and examining economic factors that differ across populations and geographic locations rather than those that are similar.

Our review aims to support systemic change by providing an overview of key classification systems complete with highlights and caveats of these systems, which is accompanied by the creation of a crosswalk tool that maps similarities between each system. In addition, we provide a synopsis of methods for visualizing PhD career outcomes, as well as tools for automating or outsourcing the tracking of this data, thus enabling institutions to choose from a variety of system(s) that work best for them. Transforming graduate and postdoctoral training and shaping the future of the research enterprise and broader economy is reliant upon informing our educators, the workforce, and our trainees about the many impactful career options pursued by graduate students and postdoctoral scholars.

Being able to reliably visualize PhD career outcomes from a large number and varying types of institutions across the country can paint a clear picture for policymakers when it comes to the makeup of the workforce and where they need to intervene, whether this means targeting specific types of universities to help them with federal funding, targeting populations that have been traditionally under-represented to assist with recruitment and retention into the pipeline, or focusing on specific disciplines or parts of the country where the workforce is not as robust as it could be. The federal government can systematically analyze this data to understand the state of the research workforce in order to consider programs in the executive branch or legislative language that can support those with historically fewer opportunities to facilitate their success in the research workforce.^44^ The success of these initiatives will depend upon the academic community joining together to identify, prioritize, and advocate for actionable policy recommendations that government systems can implement to foster the development of a diverse, robust, and innovative research workforce that will have the capability to rise to the challenges of our time.

## Supplementary Material

Data sheet 1

Data sheet 2

The Supplementary material for this article can be found online at: https://www.frontiersin.org/articles/10.3389/feduc.2025.1462887/full#supplementary-material

## Footnotes

1
http://rescuingbiomedicalresearch.org/

2
https://commonfund.nih.gov/workforce

3
https://nglscoalition.org/

4
https://gs.ucdenver.edu/fobgapt2/main.php

5
https://www.historians.org/wherehistorianswork

6
https://www.niehs.nih.gov/careers/research/fellows/alumni-outcomes/index.cfm

7
https://osf.io/dwnrk/

8
https://ncses.nsf.gov/surveys/earned-doctorates/2022#qs

9
https://www.nsf.gov/statistics/

10
https://ncses.nsf.gov/pubs/nsf21308/report/postgraduation-trends#first-postgraduate-position

11
https://www.nsf.gov/statistics/srvydoctoratework/#qs

12
https://www.bls.gov/soc/2018/#classification

13
https://www.nsf.gov/statistics/srvydoctoratework/#tabs-2&rSR&qs&sd&tabs-2&micro&profiles&tools

14
https://cgsnet.org

15
https://cgsnet.org/project/understanding-phd-career-pathways-for-program-improvement

16
https://grad.wisc.edu/career-pathways/

17
https://www.naceweb.org/job-market/graduate-outcomes/first-destination/class-of-2019/interactive-dashboard/

18
https://www.naceweb.org/job-market/graduate-outcomes/first-destination/

19
https://www.naceweb.org/uploadedfiles/pages/advocacy/first-destination-survey-standards-and-protocols-advanced.pdf

21
https://www.naceweb.org/uploadedfiles/files/2021/publication/free-report/first-destinations-for-the-class-of-2019.pdf

22
http://rescuingbiomedicalresearch.org/rbr-actions/work-in-progress/

23
http://tracephd.com/about-trace/

24
http://tracemcgill.com/wp-content/uploads/2021/03/TRaCE-McGill-QA-Report-Full-Version.pdf

25
http://tracemcgill.com/wp-content/uploads/2021/03/TRaCE_McGill_2103023-1.pdf

26
http://outcomes.grad.ubc.ca/

27
https://outcomes.grad.ubc.ca/methodology.html

28
https://www.statcan.gc.ca/eng/subjects/standard/cip/2000/index

29
https://www.sgs.utoronto.ca/about/explore-our-data/10000-phds-project/

30
https://www.aau.edu/sites/default/files/AAU-Files/PhD/Project-Summaries-02.22.19.pdf

31
https://www.census.gov/naics/

32
https://www.onetonline.org/

33
https://academicanalytics.com/

34
https://grad.tamu.edu/academics/graduate-professional-data/cngls

35
https://www.mla.org/

36
https://mlaresearch.mla.hcommons.org/2015/02/17/where-are-they-now-occupations-of-1996-2011-phd-recipients-in-2013-2/

37
https://s3.amazonaws.com/aspph-wp-production/app/uploads/2015/07/ASPPH_Graduate_Employment_Pilot_Project_Report_May2015.pdf

38
https://www.klipfolio.com/resources/articles/what-is-data-visualization

39
https://depictdatastudio.com/charts/

40
https://github.com/ft-interactive/chart-doctor/blob/master/visual-vocabulary/poster.png

41
https://extremepresentation.typepad.com/blog/2007/11/index.html

42
https://ilostat.ilo.org/methods/concepts-and-definitions/classification-occupation/

43
https://www.esf.org/fileadmin/user_upload/esf/MO_Developping_EU-careers_Report_2012.pdf

44
https://www.gao.gov/improving-federal-programs-through-data-and-evidence

## References

[R1] Abdous M, and How AI Is shaping the future of Higher Ed. (2023). Available at: https://www.insidehighered.com/views/2023/03/22/how-ai-shaping-future-higher-ed-opinion (Accessed December 11, 2023).

[R2] Alberts B, Kirschner MW, Tilghman S, and Varmus H (2014). Rescuing US biomedical research from its systemic flaws. Proc. Natl. Acad. Sci. USA 111, 5773–5777. doi: 10.1073/pnas.1404402111 24733905 PMC4000813

[R3] Allum JR, Kent JD, and McCarthy MT (2014). Understanding PhD career pathways for program improvement: a CGS report. Washington, DC: Council of Graduate Schools.

[R4] Baas T, Brandt P, Brown A, Chalkley R, Collins TRL, Fryer A, Emerging best practices for tracking trainees and for data utilization. In 2018 AAMC GREAT Conference. Atlanta, GA: United States: GREAT Group Annual Meeting 2018 (2018).

[R5] Bankston A, Ralston A, Ly J, and Singh H (2023). Development and outcomes of an international certificate program in science policy and advocacy for STEM PhD students and postdocs. bioRxiv Available at: http://biorxiv.org/content/early/2023/11/27/2023.11.26.568726.abstract (Accessed December 11, 2023).

[R6] Benderly BL (2018). A trend toward transparency for Ph.D. career outcomes? Science Careers Available at: https://www.science.org/content/article/trend-toward-transparency-phd-career-outcomes (Accessed December 11, 2023).

[R7] Bhombe A, Walukar K, Thakare Y, and Kamble S (2019). Comparative analysis of two BI tools: Micro strategy and tableau. (October 1, 2019). Proceedings of International Conference on Advancements in Computing & Management (ICACM), 559–565. doi: 10.2139/ssrn.3462539

[R8] Blank R, Daniels RJ, Gilliland G, Gutmann A, Hawgood S, Hrabowski FA, (2017). A new data effort to inform career choices in biomedicine. Science 358, 1388–1389. doi: 10.1126/science.aar4638 29242335

[R9] Brown AM, Meyers LC, Varadarajan J, Ward NJ, Cartailler J-P, Chalkley RG, (2023). From goal to outcome: analyzing the progression of biomedical sciences PhD careers in a longitudinal study using an expanded taxonomy. FASEB Bioadv 5, 427–452. doi: 10.1096/ba.2023-00072 37936923 PMC10626162

[R10] Clark E (2023). Unveiling the dark side of artificial intelligence in the job market: Forbes Available at: https://www.forbes.com/sites/elijahclark/2023/08/18/unveiling-the-dark-side-of-artificial-intelligence-in-the-job-market/?sh=8d867dc6652f (Accessed December 11, 2023).

[R11] Collins TR, Layton RL, MacDonald JE, Ramadoss D, Tessel MA, and Wheeler R Institutional graduate career outcomes database 2020. (2020). doi: 10.17605/OSF.IO/TY42S

[R12] Collins TR, Layton RL, Ramadoss D, MacDonald JE, Wheeler R, Bankston A, (2021). Making strides in doctoral-level career outcomes reporting: surveying the landscape of classification and visualization methodologies and creating a crosswalk tool. OSF doi: 10.17605/OSF.IO/97A5Z PMC12671987 41341269

[R13] Collins T, Ramadoss D, Layton RL, MacDonald J, Wheeler R, Bankston A, (2021). Making strides in doctoral-level career outcomes reporting: surveying the landscape of classification and visualization methodologies and creating a crosswalk tool. doi: 10.17605/OSF.IO/DWNRK PMC12671987 41341269

[R14] Cuzzocrea A, and Mansmann S (2009). “OLAP visualization: models, issues, and techniques” in Encylclopedia of data Warehouseing and mining, second edition (IGI Global), 1439–1446.

[R15] Feig AL, Robinson L, Yan S, Byrd M, and Mathur A (2016). Using longitudinal data on career outcomes to promote improvements and diversity in graduate education. Change 48, 42–49. doi: 10.1080/00091383.2016.1247582 28804143 PMC5551686

[R16] Forsythe E, Kahn LB, Lange F, and Wiczer DG. Evidence from vacancy postings and UI claims. Vol. 27061, NBER Working Paper. (2020). Available at: http://www.nber.org/papers/w27061 (Accessed December 11, 2023). 10.1016/j.jpubeco.2020.104238 PMC7357497 32834178

[R17] Hart J, and McKinney CC (2020). An institutional analysis of graduate outcomes reveals a contemporary workforce footprint for biomedical master’s degrees. PLoS One 15:e0243153. doi: 10.1371/journal.pone.024315344 33284826 PMC7721154

[R18] Jonker L (2016). Ontario’s PhD graduates from 2009: Where are they now? Toronto: Higher Education Quality Council of Ontario. 1–32.

[R19] Krasna H, Czabanowska K, Beck A, Cushman LF, and Leider JP (2021a). Labour market competition for public health graduates in the United States: a comparison of workforce taxonomies with job postings before and during the COVID-19 pandemic. Int. J. Health Plann. Manag 36, 151–167. doi: 10.1002/hpm.3128 PMC8014097 33625747

[R20] Krasna H, Czabanowska K, Jiang S, Khadka S, Morita H, Kornfeld J, (2020). The future of careers at the intersection of climate change and public health: what can job postings and an employer survey tell us? Int. J. Environ. Res. Public Health 17:1310. doi: 10.3390/ijerph17041310 32085475 PMC7068354

[R21] Krasna H, Gershuni O, Sherrer K, and Czabanowska K (2021b). Postgraduate employment outcomes of undergraduate and graduate public health students: a scoping review. Public Health Rep 136, 795–804. doi: 10.1177/0033354920976565 33673774 PMC8579388

[R22] Kuhn T (1962). The structure of scientific revolutions. Chicago: University of Chicago Press.

[R23] Langin KUS (2020). faculty job market tanks. Science 370, 272–273. doi: 10.1126/science.370.6514.272 33060339

[R24] Lenzi RN, Korn SJ, Wallace M, Desmond NL, and Labosky PA (2020). The NIH “BEST” programs: institutional programs, the program evaluation, and early data. FASEB J. 34, 3570–3582. doi: 10.1096/fj.201902064 31960495

[R25] Malloy J, Young L, and Berdahl L (2021). Oversupply: the system is the problem: Inside Higher Edi Available at: https://www.insidehighered.com/advice/2021/06/22/how-phd-job-crisis-built-system-and-what-can-be-done-about-it-opinion (Accessed December 11, 2023).

[R26] Mathur A, Brandt P, Chalkley R, Daniel L, Labosky P, Stayart C, (2018a). Evolution of a functional taxonomy of career pathways for biomedical trainees. J. Clin. Transl. Sci 2, 63–65. doi: 10.1017/cts.2018.22 30364657 PMC6197487

[R27] Mathur A, Cano A, Kohl M, Muthunayake NS, Vaidyanathan P, Wood ME, (2018b). Visualization of gender, race, citizenship and academic performance in association with career outcomes of 15-year biomedical doctoral alumni at a public research university. PLoS One 13, 13:e0197473. doi: 10.1371/journal.pone.0197473 29771987 PMC5957427

[R28] Mathur A, Chow CS, Feig AL, Kenaga H, Moldenhauer JA, Muthunayake NS, (2018c). Exposure to multiple career pathways by biomedical doctoral students at a public research university. PLoS One 13:e0199720. doi: 10.1371/journal.pone.0199720 29933412 PMC6014666

[R29] Mathur A, Lean SF, Walker NV, Kohl M, Ziyad M, Cano A, (2018d). Career outcomes for STEM, social and behavioral sciences and education doctoral alumni. Ageing Sci. Ment. Health Stud 2, 1–33. doi: 10.31038/ASMHS.2018233

[R30] McAlpine L, Skakni I, and Inouye K (2021). PhD careers beyond the traditional: integrating individual and structural factors for a richer account. Eur. J. High. Educ 11, 365–385. doi: 10.1080/21568235.2020.1870242

[R31] Mikell JG How to convey the benefits of higher education to government and policymakers. (2023). Available at: https://www.timeshighereducation.com/campus/how-convey-benefits-higher-education-government-and-policymakers (Accessed December 11, 2023).

[R32] Murphy SA (2013). Data visualization and rapid analytics: applying tableau desktop to support library decision-making. J. Web Librariansh. 7, 465–476. doi: 10.1080/19322909.2013.825148

[R33] National Academies of Sciences, Engineering, and Medicine (2018a). Graduate STEM education for the 21st century. eds. Leshner A and Scherer L (Washington, DC: The National Academies Press). Available at: https://www.nap.edu/catalog/25038/graduate-stem-education-for-the-21st-century (Accessed December 11, 2023).

[R34] National Academies of Sciences, Engineering, and Medicine (2018b). The next generation of biomedical and behavioral sciences researchers: breaking through. eds. Daniels R and Beninson L (Washington, DC: The National Academies Press). Available at: https://www.nap.edu/catalog/25008/the-next-generation-of-biomedical-and-behavioral-sciences-researchers-breaking (Accessed December 11, 2023). 29664586

[R35] National Academy of Sciences, National Academy of Engineering, Institute of Medicine (2014). The postdoctoral experience revisited. Washington, DC: The National Academies Press Available at: https://www.nap.edu/catalog/18982/the-postdoctoral-experience-revisited (Accessed December 11, 2023). 25590106

[R36] Neumann R, and Tan KK (2011). From PhD to initial employment: the doctorate in a knowledge economy. Stud. High. Educ 36, 601–614. doi: 10.1080/03075079.2011.594596

[R37] Oliver K, Lorenc T, and Innvær S (2014). New directions in evidence-based policy research: a critical analysis of the literature. Health Res. Policy Syst 12:34. doi: 10.1186/1478-4505-12-34 25023520 PMC4107868

[R38] Overseas Development Institute. 10 things to know about how to influence policy with research. (2017). Available at: https://commonslibrary.org/10-things-to-know-about-how-to-influence-policy-with-research/(Accessed December 11, 2023).

[R39] Panchanathan S (2023). STEM must meet people where they are. Science 380:563. doi: 10.1126/science.adi5538 37167397

[R40] Plepys CM, Krasna H, Leider JP, Burke EM, Blakely CH, and Magaña L (2021). First-destination outcomes for 2015–2018 public health graduates: focus on employment. Am. J. Public Health 111, 475–484. doi: 10.2105/AJPH.2020.306038 33476234 PMC7893365

[R41] Porter S, Mol L, Locher J, and Johnston M. UBC PhD career outcomes graduates from 2005–2013. (2017). Available at: http://outcomes.grad.ubc.ca/docs/UBC_PhD_Career_Outcomes_April2017.pdf (Accessed December 11, 2023).

[R42] Reithmeier R, O’Leary L, Zhu X, Dales C, Abdulkarim A, Aquil A, (2019). The 10,000 PhDs project at the University of Toronto: using employment outcome data to inform graduate education. PLoS One 14, 14:e0209898. doi: 10.1371/journal.pone.0209898 30650157 PMC6334897

[R43] Rohde J, France J, Benedict B, and Godwin A Exploring the early career pathways of degree holders from biomedical, environmental, and interdisciplinary/multidisciplinary engineering. (2020). doi: 10.18260/1-2--34646

[R44] Russ DE, Ho K-Y, Colt JS, Armenti KR, Baris D, Chow W-H, (2016). Computer-based coding of free-text job descriptions to efficiently identify occupations in epidemiological studies. Occup. Environ. Med 73, 417–424. doi: 10.1136/oemed-2015-103152 27102331 PMC4871757

[R45] Sauermann H, and Roach M (2012). Science PhD career preferences: levels, changes, and advisor encouragement. PLoS One 7:e36307. doi: 10.1371/journal.pone.0036307 22567149 PMC3342243

[R46] Silva EA, Des Jarlais C, Lindstaedt B, Rotman E, and Watkins ES (2016). Tracking career outcomes for postdoctoral scholars: a call to action. PLoS Biol 14:e1002458. doi: 10.1371/journal.pbio.1002458 27152650 PMC4859534

[R47] Silva EA, Mejía AB, and Watkins ES (2019). Where do our graduates go? A tool kit for tracking career outcomes of biomedical PhD students and postdoctoral scholars. CBE Life Sci. Educ 18:le3. doi: 10.1187/cbe.19-08-0150 31702952 PMC8727058

[R48] Sinche M, Layton RL, Brandt PD, O’Connell AB, Hall JD, Freeman AM, (2017). An evidence-based evaluation of transferrable skills and job satisfaction for science PhDs. PLoS One 12, 1–16. doi: 10.1371/journal.pone.0185023 PMC5607200 28931079

[R49] Stayart CA, Brandt PD, Brown AM, Dahl T, Layton RL, Petrie KA, (2020). Applying inter-rater reliability to improve consistency in classifying PhD career outcomes [version 2; peer review: 2 approved]. F1000Res 9:8. Available at: http://openr.es/k1e (Accessed December 11, 2023). 32089837 10.12688/f1000research.21046.1 PMC7014580

[R50] Swafford E, and Ruediger D. Every historian counts: A new AHA database analyzes careers for PhDs. Perspectives on history. (2018); Available at: https://www.historians.org/publications-and-directories/perspectives-on-history/september-2018/every-historian-counts-a-new-aha-database-analyzes-careers-for-phds (Accessed December 11, 2023).

[R51] Tilghman S, Rockey S, Degen S, Forese L, and Ginther D. Biomedical research workforce. (2012); Available at: https://acd.od.nih.gov/documents/reports/Biomedical_research_wgreport.pdf (Accessed December 11, 2023).

[R52] Van Wart A, Djorić D, D’Silva NM, Layton R, Hardy L, Suelzer E, (2023). An emerging field: an evaluation of biomedical graduate student and postdoctoral education and training research across seven decades. PLoS One 18:e0282262. doi: 10.1371/journal.pone.0282262 37490486 PMC10368290

[R53] Wapman KH, Zhang S, Clauset A, and Larremore DB (2022). Quantifying hierarchy and dynamics in US faculty hiring and retention. Nature 610, 120–127. doi: 10.1038/s41586-022-05222-x 36131023 PMC9534765

[R54] Wenqin S, Yao G, Bin Z, and Jin J (2018). Academia or enterprises: gender, research outputs, and employment among PhD graduates in China. Asia Pac. Educ. Rev 19, 285–296. doi: 10.1007/s12564-018-9538-5

[R55] Xu H, Gilliam RST, Peddada SD, Buchold GM, and Collins TRL (2018). Visualizing detailed postdoctoral employment trends using a new career outcome taxonomy. Nat. Biotechnol 36, 197–202. doi: 10.1038/nbt.4059 29334368 PMC5872819

[R56] Yang Y, and Fumasoli T (2024). Occupational choice, satisfaction and success of PhD graduates in East Asia and the west: a systematic review. High. Educ. Q. 78, 307–332. doi: 10.1111/hequ.12490

