# Supplementary material for: Making strides in doctoral-level career outcomes reporting: a review of classification and visualization methodologies in graduate education
Source: Front Educ (Lausanne). Author manuscript; Available in PMC 2025 Dec 3. (PMC12671987; doi:10.3389/feduc.2025.1462887)
Supplement: Data sheet 1 [file NIHMS2110404-supplement-Data_sheet_1.pdf]

Supplemental Material 2: Resources, Tools, & Coalitions[1]

Herein we describe a variety of coalitions and collaborative efforts whose aim is to facilitate others' ability to collect and disseminate information on the career outcomes of graduate students and postdoctoral scholars. We also describe some key resources (often available for a fee) that can further aid institutions in managing, analyzing, and reporting these data.

1 Resources & Tools: The **Institute for Research on Innovation in Science (IRIS)** is a consortium of universities whose members pay to receive access[2] to an IRB-approved data repository that is housed at the University of Michigan. Member institutions share alumni data with IRIS[3], which is de-identified and combined with datasets from other sources (e.g., the U.S. Census Bureau, public federal sources, private sector sources) to obtain a more complete picture of both the career outcomes of alumni as well as the social and economic impacts of investment into the domestic U.S. scientific research enterprise. A limitation of the domestic focus is that many of the repositories used to combine data are only available within the U.S. (some Canadian data sources), precluding collection of international fellows' data.

1.1 Steppingblocks is an education and workforce data analytics provider which entered into a unique partnership with IRIS[4]. Steppingblocks' core technology is to structure public data from a multitude of sources, and to employ machine learning to de-duplicate, clean, and categorize data down to the individual level[5]. With the linkage of IRIS and Steppingblocks' data together, a wealth of information may be obtained, such as award funding history, funding timeframe, employer, salary, etc. This information can be distributed to members as specifically tailored reports to meet institutional needs. IRIS members (membership cost) can also request a download of all underlying data in tabular form so that data obtained from IRIS can be combined with other university-specific datasets. A key aspect of IRIS membership is assistance with publicity to highlight accomplishments of the research enterprise. A press release accompanies each member institutions' report.

1.2 Academic Analytics is a company originally created to collect and analyze data on institutions' research[6]. Academic Analytics now also collects graduate and postdoctoral outcomes data. Institutions that hire the company provide them with a list of graduates (names and the year or date of graduation) for Academic Analytics to track, via internet searches, the location of their graduates' employers. Most subscribing institutions provide 10 years of graduate names and contact information and receive collected data for those individuals identified. The company provides person-level information, employer name, position title, and three types of classifications based on the following taxonomies:

- Coalition for Next Generation Life Science (CNGLS)[7]
- North American Industry Classification Systems (NAICS)/Standard Industrial Classifications (SIC) & Standard Occupational Code (SOC) Listings[8]
- AAUDE[9]

All classifications are done systematically following a logic tree. Data are updated every year, with a rolling 10-year window. Visualizations are simple, and done in Tableau, displaying basic information on career types, race, gender, ethnicity, citizenship, etc.

1.3 Lightcast is a company that was formed by the merging of **Burning Glass Technologies** and **Economic Modeling Specialists International (EMSI).** For a fee, it offers a wide range of services, including real-time information on the labor market (such as how it was affected by COVID-19, e.g. Forsythe et al., 2020; Krasna et, al., 2021) by scanning

[1] NOTE: In supplemental files, websites appear as footnotes, while in-text citations refer to the primary manuscript's reference section.
[2] https://iris.isr.umich.edu/membership/join/
[3] https://iris.isr.umich.edu/research-data/
[4] https://www.steppingblocks.com
[5] https://www.youtube.com/watch?v=K6sh0GpOdT4
[6] https://academicanalytics.com
[7] http://nglscoalition.org/progress/
[8] https://www.nsca.org/naicssoc-codes/
[9] https://www.aaude.org/

millions of job ads across the globe and conducting in-depth data analytics to determine what skills are sought after and where certain jobs are located[10]. Lightcast also has a specific product solution entitled “Alumni Outcomes Tracking” which offers a variety of options--from tracking software to surveys on alumni sentiment. Outcomes are classified by SOC/O*NET code, with employer type classified by NAICS codes. Lightcast will provide information such as the following: 1) an overview of career outcomes that is filterable; 2) a focused snapshot of employment outcomes in specific programs, 3) top employers, 4) alumni by location, 5) job titles, 6) estimated salaries. Further assistance with analytics is available, such as benchmarking to national standards or allowing students to explore potential wage earnings as a function of career outcome.

Several **Tools for Automating or Crosswalking Standard Job Codes** have been developed which allow matching of large-scale text, especially from job postings data, to SOC codes. This includes the NIH’s Standardized Occupation Coding for Computer-assisted Epidemiological Research or SOCcer tool[11] (Russ et al., 2016). This tool has also been used to analyze sub-sectors of the job market by cross-referencing data scraped from job posting aggregation sites like Indeed.com (Krasna et al., 2020).

Other tools exist which can “crosswalk” between CIP codes and SOC codes[12]; and between the US Department of Labor’s SOC codes and international codes[13] such as ISCO-08[14], allowing international comparisons. A similar international comparison tool has been developed in Canada, Codage Assisté des Professions et Secteurs d’activité[15]. A tool from the CDC can automate assignment of NAICS or SOC codes as well as crosswalk between different versions of each[16].

2 Coalitions

2.1 NIH Broadening Experiences in Scientific Training (NIH BEST) Consortium (Lenzi et al., 2020)**, Rescuing Biomedical Research (RBR)[17], and Future of Bioscience Graduate and Postdoctoral Training (FOBGAPT)[18]** spearheaded efforts to develop, refine, and adopt a unified, 3-tiered career outcomes taxonomy divided by workforce sector, career type, and job function, later named the Unified Career Outcomes Taxonomy and broadly implemented by the member institutions of CNGLS.

2.2 The **Coalition for Next Generation Life Science (CNGLS)** formed in 2017 to address calls for greater transparency in graduate and postdoctoral training. Since formation, coalition membership has expanded from 10 founding institutions to 52 as of 2023, according to the CNGLS website. Member institutions agree to publish career outcomes data for PhD and postdoctoral alumni according to the top two tiers of the UCOT 2017 taxonomy—workforce sector and career type[19].

2.3 The **Council of Graduate Schools (CGS)** launched a PhD Career Pathways project to help institutions understand the professional aspirations and career pathways of PhDs. As of 2023, 75 Doctoral Institutions have participated in the project. Several “Research in Brief” articles have been published, highlighting aggregate results from the survey[20].

2.4 The **American Association of Universities (AAU)** began an initiative in 2014 to encourage institutions to collect student employment outcomes data. They have since formally launched the AAU PhD Education Initiative, whose goal is

---

[10] https://lightcast.io/
[11] https://soccer.nci.nih.gov/
[12] https://nces.ed.gov/ipeds/cipcode/Files/IES2020_CIP_SOC_Crosswalk_508C.pdf
[13] https://ilostat.ilo.org/methods/concepts-and-definitions/classification-occupation/
[14] https://www.bls.gov/soc/isco_soc_crosswalk_process.pdf
[15] https://host.credim.u-bordeaux.fr/caps-fr/Rechercher.aspx
[16] https://csams.cdc.gov/nioccs/Default.aspx
[17] http://rescuingbiomedicalresearch.org/
[18] https://gs.ucdenver.edu/fobgapt2/pdf/FOBGAPT2_whitepaper_final.pdf
[19] https://nglscoalition.org/
[20] https://cgsnet.org/resources/data-infographics

to make data about PhD career pathways and employment trends widely available. The AAU has also collated and summarized lists of career outcome tracking efforts[21-22].

2.5 The National Academies of Science, Engineering, and Medicine (NASEM) released two consensus reports in 2018 about graduate and postdoctoral STEM education and training: "The Next Generation of Biomedical and Behavioral Sciences Researchers: Breaking Through"[23] and "Graduate STEM Education for the 21st Century"[24]. The first study made a key recommendation for institutions to collect and disseminate career outcomes data using common standards and definitions. It suggests the NIH incentivize such data collection by making it a requirement. The second study recommended transparency of career outcomes in order for current and prospective students to be able to make educated choices and to enable institutions to make effective adjustments. It also urged the need for standardization, transparency, and accessibility.

2.6 Future of Research members contributed to the NASEM report on the biomedical research enterprise (Breaking Through), and participated in a workshop on mentoring (The Science of Effective Mentorship in STEM) with discussions incorporated into the NASEM report. The group has also organized workshops and built a webpage, "Tracking Career Outcomes at Institutions"[25], collating a list of U.S. institutions and organizations that have collected and published career outcomes data.

2.7 The **American Association of Medical Colleges (AAMC)** released the National M.D.-PhD Program Outcomes Study in April 2018[26], covering the career paths of physician-scientists. AAMC representatives also worked with RBR and NIH BEST to adopt a unified career outcomes taxonomy. AAMC **Group on Graduate Research Education and Training (GREAT Group)** additionally organizes annual conferences to promote and discuss graduate education, professional development, and training related topics including career outcomes tracking.

2.8 National Institute of General Medical Science (NIGMS) issued an updated Institutional Predoctoral Training Grant Funding Opportunity Announcement in 2017, which features new guidelines for predoctoral training grant (T32) applications. Among other changes, applicants will need to provide information about career outcomes, career exploration opportunities, and professional skills development for their trainees[27-28].

2.9 The **National Institute of Environmental Health Sciences (NIEHS)** extramural division developed a system entitled 'CareerTrac'[29] to enable tracking of trainees' employment outcomes and accomplishments over time from laboratories receiving NIH funding. It was the first system of its kind to be developed at an NIH institute, and is also used by the Fogarty International Center, NIGMS, National Cancer Institute, and the National Institute of Diabetes and Digestive and Kidney Diseases (NIDDK). A poster describing outcomes from those previously on T32 training grants has been presented[30].

2.10 NORC at the University of Chicago: Progress and Pitfalls in Monitoring Doctoral Degree Holders' Career Paths is an NSF grant-funded project that supports four main activities: a web-based national survey of graduate deans in fall 2018 to assess current practices of monitoring graduates' careers; a set of focus groups of graduate deans in December

---

[21] https://www.aau.edu/sites/default/files/AAU-Files/PhD/10.18.18_Multi-Institutional_Efforts.pdf
[22] https:/www.aau.edu/sites/default/files/AAU-Files/PhD/Project-Summaries-02.22.19-1.pdf
[23] https://www.nap.edu/catalog/25008/the-next-generation-of-biomedical-and-behavioral-sciences-researchers-breaking
[24] https://www.nap.edu/catalog/25038/graduate-stem-education-for-the-21st-century
[25] http://futureofresearch.org/tracking-career-outcomes-at-institutions/
[26] https://store.aamc.org/downloadable/download/sample/sample_id/162/
[27] https://loop.nigms.nih.gov/2017/10/new-nigms-institutional-predoctoral-training-grant-funding-opportunity-announcement/
[28] https://grants.nih.gov/grants/guide/pa-files/PAR-17-341.htmlx
[29] https://careertrac.niehs.nih.gov/public/staticPage/about
[30] https://www.niehs.nih.gov/research/supported/assets/docs/a_c/careertrac_evaluating_t32_training_outcomes_508.pdf

2018 that will address guiding questions informed by the survey; a one-and-a-half-day conference in May 2019 with the goal of developing standards for collecting and reporting data on doctoral career pathways; and a multipronged dissemination of the project results[31].

2.11 The **American Psychological Association (APA)** partnered with **Economic Modeling Specialists International (EMSI)** (now **Lightcast**) for tracking and analyzing the post-graduation outcomes of psychologists, which they expect will provide a wealth of information useful for creating career development resources, as well as determining the impacts of the coronavirus pandemic on the psychology workforce. In this study, APA also aims to identify the specific skill sets that psychologists are using in their careers. At present, the APA's Center for Workforce Studies has a wealth of information on the psychology workforce, such as the one found on their data tools outlining the types of activities that psychologists are engaged in[32].

2.12 The **Graduate Career Consortium (GCC)'s Outcomes Committee** collated information on the publicly available graduate and postdoctoral alumni career outcomes from each of its member institutions. Approximately 63% of member institutions publicly report on their career outcomes in a quantitative manner in 2020. A comprehensive database of these outcomes was released[33] and will be updated by the Graduate Career Consortium as able (Collins, T.R. et al., 2020).

---

[31] https://www.norc.org/content/dam/norc-org/pdfs/Progress and Pitfalls in Tracking U.S. Doctoral Career Paths NORC.pdf

[32] https://www.apa.org/workforce/data-tools/careers-psychology

[33] https://doi.org/10.17605/OSF.IO/97A5Z
