# Supplementary material for: Making strides in doctoral-level career outcomes reporting: a review of classification and visualization methodologies in graduate education
Source: Front Educ (Lausanne). Author manuscript; Available in PMC 2025 Dec 3. (PMC12671987; doi:10.3389/feduc.2025.1462887)
Supplement: Data sheet 2 [file NIHMS2110404-supplement-Data_sheet_2.pdf]

Supplemental Material 1: Visualization Platforms and Types[1]

Herein we list platforms/software that are widely used to display career outcomes data and stories.

1 Visualization Platforms

1.1 Tableau: Tableau software supports popular visualizations such as tables, charts, maps, time series, etc. Despite its simplicity, Tableau is a powerful tool with advantages including integrating querying, exploration, and visualization of data into a single process (Murphy, 2013). It is shown to have high performance with large data sets and can connect to a varied set of data sources (Bhombe et al., 2015). Tableau is capable of providing *ad-hoc* analyses and has provisions to analyze data offline. A potential limiting feature is that Tableau is constrained to generating visual presentation grids with uniform and limited granularity and dimensionality (Cuzzocrea and Mansmann, 2009). Nonetheless, when representing outcomes data, this uniformity may be desirable if one wants to compare outcomes between institutions.

Some selected examples of using Tableau to visualize career outcomes data at various institutions include Stanford University[2], Johns Hopkins University[3], the University of California-San Francisco[4], and the University of North Carolina at Chapel Hill[5]. Institutions vary in how they visualize data within Tableau, yet a significant proportion tend to display data in bar charts and tables, with dropdown filters available to further explore and parse the data.

1.2 NIEHS custom-made R platform: NIEHS built a custom data platform[6] using open source R language[7], RStudio[8], and the R Shiny[9] package that allows users to build interactive web apps and dashboards straight from R. A software add-on known as Plotly[10], an R package for creating interactive web-based graphs via the open source JavaScript graphing library, was also incorporated into the interface to increase the level of interactivity with the graphics. The platform functions by reading an Excel file and presenting data in a standardized output format. An advantage of using this platform is that it provides full control of how data are presented—including in the nature and types of graphics shown. The platform also allows users to easily present binned data (e.g., 5-year bins, or any desired bin size), which can allow one to avoid presenting data with small sample sizes—thus addressing potential privacy concerns while also making data interpretation more robust. The graphing possibilities using R are plentiful: basic diverging bar charts; donut charts; point-range charts; box-and-whisker plots; Sankey graphs; bubble plots overlaid with heat maps; directional chord diagrams, etc. These visualizations could showcase: a) how demographics change over time; b) how each of three taxonomic tiers relate to each other; c) how country of origin relates to location of job employment; d) how training times differ over time and according to career outcome; e) how outcomes differ as a function of country of origin while simultaneously overlaying either gender differences, training time differences, job location differences, and more.

To date, NIEHS has adapted this platform for several universities' internal use, and has included key additions such as whether an individual was on a T32 training grant or received their own funding. In order for another institution to use the platform, they need only to format their data in a pre-specified manner (download example "Source Data" file

---

[1] NOTE: In supplemental files, websites appear as footnotes, while in-text citations refer to the primary manuscript's reference section.

[2] https://tableau.stanford.edu/t/IRDS/views/StanfordPhDAlumniEmployment/StanfordPhDAlumniEmploymentDashboard?:embed_code_version=3&:embed=y&:loadOrderID=0&:display_spinner=no&:display_count=n&:showVizHome=n&:origin=viz_share_link

[3] https://provost.jhu.edu/education/graduate-and-professional-education/cngls/life-science-career-outcomes/

[4] https://graduate.ucsf.edu/program-statistics

[5] https://bbsp.unc.edu/professional-development/career-outcomes/

[6] https://www.niehs.nih.gov/careers/research/fellows/alumni-outcomes/index.cfm

[7] https://www.r-project.org/

[8] https://posit.co/download/rstudio-desktop/

[9] https://shiny.rstudio.com/

[10] https://plotly.com/r/getting-started/

located at the bottom of the NIEHS Alumni Outcomes Dashboard[11]). The R platform can then read the Excel file and instantly produce the dashboard as an output. If an institution's data are not formatted in the manner shown, they can collaborate with NIEHS to adapt the platform to their needs or work within their own institution to modify the R-code for their own purposes. The code, as well as a comprehensive handbook[12] containing instructions on how to use and modify the code, can be found for both the original dashboard version[13] and for the updated version[14].

1.4 Microsoft Excel: MS Excel is a ubiquitous, low-cost, and easy-to-use tool typically used for basic data analytics. It affords a first step to data visualization, with multiple types of first-order charts. Basic charts available include column, line, pie, bar, area, and scatter plots, and for more sophisticated visualizations, stock, surface, bubble, donut and radar charts are available. Some variability exists within each chart type, such as 2-dimensional vs. 3-dimensional visualization, use of color, etc. Excel visualizations for PhD career outcomes data include divergent stacked bars, word clouds, and Sankey diagrams (e.g., Oregon Health & Sciences University[15]; and tutorial[16]. There are a number of training resources for improving Excel visualization skills; one such example can be found here (Depict Data Studio[17]). For those desiring more advanced graphic functionalities, a Poweruser plugin[18] can be installed that will enable one to create more complex graphics within Excel, such as Sankey diagrams.

1.5 Career Service Management (CSM) Platforms: Three of the most popular CSMs on the market are Symplicity[19], Handshake[20], and 12Twenty[21]—each of which offers modules that permit institutions to track, measure, and visually present their first destination survey outcomes. Symplicity, Handshake, and 12Twenty allow institutions to develop and launch first destination surveys tailored to their needs and, in the case of Symplicity, integrate the NACE First Destination Survey (FDS) into their CSM dashboard. These are user-friendly systems that are easy to navigate—especially for those who lack data skills, as the data analysis is automated by the CSM.

The integration of first destination outcomes into CSMs is relatively recent and reflects the growing interest of educational institutions to provide statistical evidence of their students' success as well as to potentially provide prospective students with data pertaining to their programs to help them make informed choices. While the data collected through the CSM are frequently presented on outward-facing webpages, the integration of first destination survey outcomes in the CSM permits current students and alumni to identify career paths, industry trends, and alumni employment locations associated with their respective programs.

First Destination Survey questions may be integrated into CSM, or data collected through surveys managed outside of the CSM may be uploaded into them following the CSM-designated method. Users (students and administrators alike) can select from predetermined and/or customized fields to view visualizations of the data. FDS outcomes may be included as part of a platform or as add-ons depending on the particular CSM.

1.6 Microsoft PowerBI: Power BI[22] is a business intelligence (BI) analytics service by Microsoft with both cloud- and desktop-based interfaces. Data can be imported into Power BI from a variety of sources such as Microsoft Excel, MailChimp, Salesforce, etc. through a breadth of data connectors. Large datasets, commonly referred to as "Big Data",

---

[11] https://www.niehs.nih.gov/careers/research/fellows/alumni-outcomes/index.cfm
[12] https://github.com/nihxuh/alumni-customization/commit/17451228e31c38c8d56d9b85f54699428cae1e54
[13] https://github.com/nihxuh/alumni-shiny
[14] https://github.com/NIEHS/alumni-dashboard/
[15] https://www.ohsu.edu/sites/default/files/2020-04/OHSU%20SoM%20Outcomes%202019%20Report%2004082020.pdf
[16] https://peltiertech.com/diverging-stacked-bar-charts/
[17] https://depictdatastudio.com/visualizing-equity-in-education/
[18] https://www.powerusersoftwares.com
[19] https://www.symplicity.com/
[20] https://joinhandshake.com/career-centers/?_ga=2.134659441.1874268023.1607558995-968462549.1607558995
[21] https://www.12twenty.com/
[22] https://powerbi.microsoft.com/en-us/

can also be integrated directly into the Power BI web service, which allows for easy sharing. Power BI boasts an intuitive user interface. Once data are loaded, users can click through a variety of options to choose the desired visualization, which includes Sankey diagrams, bullet charts, aster plots, word clouds, and more. Formats for various visualizations can be easily customized, and many options are also available for interactive visualizations. More complex tasks such as joining datasets can also be easily accomplished. With both the simplicity in building visualizations and the plentiful customization options, Microsoft Power BI is generally a highly rated tool. Some selected examples of graduate-level career outcomes showcased with Microsoft Power BI include Wayne State University[23], the University of Texas System[24], and Weill Cornell Medicine[25]. NACE, as mentioned above in the taxonomies, showcases the collective outcomes of college undergraduates using Power BI[26]. Power BI Desktop is available to download for free, while other options (e.g., Power BI Pro) have subscription costs, and include features such as API embedding, peer-to-peer sharing, and support for data analysis. One limitation to note is that Power BI only functions within a Microsoft Windows environment; individuals with other operating systems must utilize a virtual Windows desktop to run Power BI.

1.7 GraphPad Prism, IBM SPSS, & SAS: Some data analysis software packages are also equipped to provide accompanying visualizations using check-box and pre-programmed options. This is especially useful for individuals without coding experience. Both Prism[27] and SPSS[28] are examples of plug-and-play software platforms that allow the use of default settings and that help guide users in choosing which statistical tests to use based on the data selected. Prism, which is oriented toward life-science examples, is especially user-friendly with tutorials and sample data sets, whereas SPSS is oriented toward complex analyses using control variables and was originally developed to support research in the social sciences. Both software packages produce useful visualizations such as graphs, box-and-whiskers plots, scatter plots, and more. SPSS visualizations are typically useful while completing analyses, but other software packages produce more customizable variations reproduced for publication-quality visualizations (e.g., R, IBM SAS[29], Prism, or other customizable visualization tools). Prism is easily customized and can create publication-quality visualizations, though some of its advanced analytics are less developed than the default options available in IBM SPSS. SAS provides innovative visualizations and robust data storage abilities, and it supports customized complex analyses. However, SAS requires coding knowledge to fully use these features. Each of these software options can provide basic analyses and basic visualization tools, so available institutional training and support may dictate adoption at the department, office, or institutional level.

1.8 Institution-Specific (Customized Platforms): Some institutions have developed homegrown solutions that are fully customized for their needs. This may be a viable option if internal institutional resources are available to assist, if the staff member responsible has skills in this area already, or if trainees at the institution can be trained to support development of the customized system. Alternatively, institutional or grant funds may be leveraged for an initial one-time investment to set up a complex customized system if its maintenance over time is possible long-term or if an ongoing contract is feasible. For instance, UBC used a Java-based (JQuery) hosted on a Google library[30-31] and Clemson University similarly used a java-based Google library[32] while Boston University previously used a Plotly Javascript library but has now moved to Microsoft PowerBI[33]. While these examples are not intended to be comprehensive, they illustrate some examples of institutions that have developed their own fully customized platforms to display their data.

---

[23] https://gradschool.wayne.edu/about/coalition-next-gen
[24] https://seekut.utsystem.edu/GradNat
[25] https://mdphd.weill.cornell.edu/alumni-and-outcomes/career-paths-our-graduates
[26] https://www.naceweb.org/job-market/graduate-outcomes/first-destination/class-of-2019/interactive-dashboard/
[27] https://www.graphpad.com/scientific-software/prism/
[28] https://www.ibm.com/products/spss-statistics
[29] https://www.sas.com/en_us/home.html
[30] https://outcomes.grad.ubc.ca/comp-cohorts.html
[31] https://developers.google.com/speed/libraries#jquery
[32] https://career.sites.clemson.edu/data_analytics/FDS_App.php?year=18_19&filters=%60degree%60=%22Doctoral%20degree%22#open
[33] https://www.bu.edu/grad/why-bu/phd-program-profiles/epidemiology/

2 Visualization Types

As with the other sections of this report, the following list of visualizations is intended to be informative but not exhaustive. While outcomes data can be visualized using basic pie charts, donut charts, bar charts, and line charts, we have chosen to include the career outcomes visualization examples that showcase data in innovative ways. A variety of resources exist to help decide which visualization can best illustrate your career outcome stories[34].

2.1 Diverging Stacked Bars: Diverging Stacked Bars can allow for visualization of changes over time, such as with career trajectory or demographics. As an example, snapshots of data for the graduate population at Oregon Health & Sciences University (OHSU)[35] are captured at specific time points after completing their PhD (e.g., 1st year, 5th year, 10th year) (Fig. 1). For each Year-Post-Graduation cohort, the graduates are represented as a bar with various colored segments—each color of which depicts the relative percentage of alumni in a particular career type, with the total of segments equaling 100% of the cohort. These cohort years are stacked on top of each other, and a divergence point is chosen. In this case, OHSU chose the divergence point as postdoctorates and tenure-track faculty. When each cohort is stacked one on top of the other, it becomes immediately apparent when the proportion in the largest career type (in this case, postdoctorates) changes across the different cohorts based on how many years have passed post-graduation. As the number of years postgraduation increases, the number of alumni in postdoctorate positions decreases, while the number in tenure-track faculty increases. Increases in other careers can also be seen, though the largest movement appears to be in the transition from postdoc to faculty. As indicated in the graphic, the raw number of PhD graduates surveyed across each cohort differs; it is important to note these differences when visualizing data so that readers can ascertain the limitations of data interpretation. In another example, diverging stacked bars are used to show how population demographics of trainees at the NIEHS (Xu et al., 2018) changed over time, from a higher percentage of males (over 60%) in the early 2000's to a more balanced, nearly 50/50 population of males and females in the 2010's. These types of graphics can be created in a number of programs such as Excel[36] and are also readily made within other software and programming languages (including Tableau, Python, R)[37].

[34] https://depictdatastudio.com/charts/
[35] https://www.ohsu.edu/sites/default/files/2020-04/OHSU%20SoM%20Outcomes%202019%20Report%2004082020.pdf
[36] http://stephanieevergreen.com/diverging-stacked-bars/
[37] https://towardsdatascience.com/diverging-bars-why-how-3e3ecc066dce

Figure 1: Using Diverging Stacked Bars to Visualize Changes in Career Outcomes Over Time.

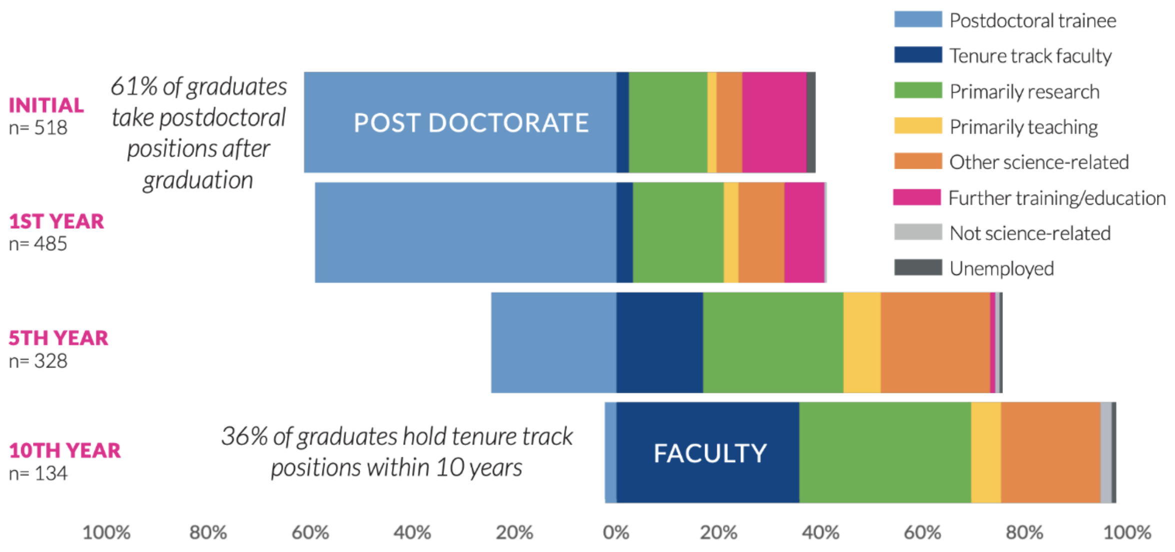

Figure 1 Caption: *Diverging stacked bars showcase the relative percentage of PhD graduates from the Oregon Health & Science University School of Medicine entering into careers at given points after graduation (initial position; 1 year after graduation; 5 years after graduation; 10 years after graduation). 36% of graduates become tenure-track faculty within 10 years after graduating. Figure provided by Allison D Fryer, Jackie Wirz and Amanda Mather, Graduate Studies School of Medicine, Oregon Health & Science University.* [38] *Reproduced with permission.)*

2.2 Sankey Diagram: A Sankey diagram allows for complex visualization of the relationships between two or more variables with multiple possible outcomes. For example, a Sankey diagram can show how individuals with degrees in different fields 'flow' into different careers upon graduation – examples include diagrams from Wayne State University (Fieg et. Al., 2016), Stanford University[39], and Purdue University (Murphy, 2013). This same type of flow has also been shown in terms of geography, in which alumni from a given country 'flow' into a career either in the same country or a different country (e.g., UBC[40]). Furthermore, a Sankey diagram can be used to emphasize relative proportions; one example from the National Institute of Environmental Health Sciences (Xu et al., 2018) visualizes three tiers of a taxonomy (Fig. 2). Taking the academic job sector (shown in red) on the far left as an example—as the viewer moves to the right visually, career outcomes in the academic sector are further divided into professional, management, tenure-track, support, non-tenure-track, and trainee job types. If one focuses on the tenure-track job type (shown in green) and continues moving to the right, one can see that the main proportion of those in this job type are conducting basic research. An interactive form of this diagram is also available[41] (see 'Relationship between categories'). A potential pitfall of using a Sankey diagram to illustrate proportions is that the data may be misinterpreted as 'flow,' meaning, for example, that someone may think an individual is moving from an academic career to a management career to a basic research career. Therefore, it is critical to clearly label the Sankey diagram when it is used in this manner. A Sankey diagram was chosen in Fig. 2 because it clearly and effectively illustrates how the career outcomes from all three tiers of a taxonomy are related to each other, which is not possible when each tier is represented separately.

Sankey diagrams can be made in R or Python[42] as well as in many of the platforms described above such as Tableau and Microsoft Power BI. They can also be created in Excel if the add-in power-user[43] is installed. Sankeymatic[44] is also a

[38] *https://www.ohsu.edu/sites/default/files/2020-04/OHSU%20SoM%20Outcomes%202019%20Report%2004082020.pdf*
[39] https://irds.stanford.edu/data-findings/phd-jobs
[40] https://outcomes.grad.ubc.ca/geographic-movement.html
[41] https://www.niehs.nih.gov/careers/research/fellows/alumni-outcomes/index.cfm
[42] https://www.data-to-viz.com/graph/sankey.html
[43] https://www.powerusersoftwares.com/
[44] http://sankeymatic.com/

helpful tool for creating a Sankey diagram that does not require coding experience or any additional external software. Users input the data into the online interface, and export the diagram as a JPEG, which can be further formatted using other software such as Adobe Illustrator or Photoshop.

Figure 2. Using a Sankey Diagram to Illustrate Career Outcomes and the Relationship Between Tiers of a Taxonomy.

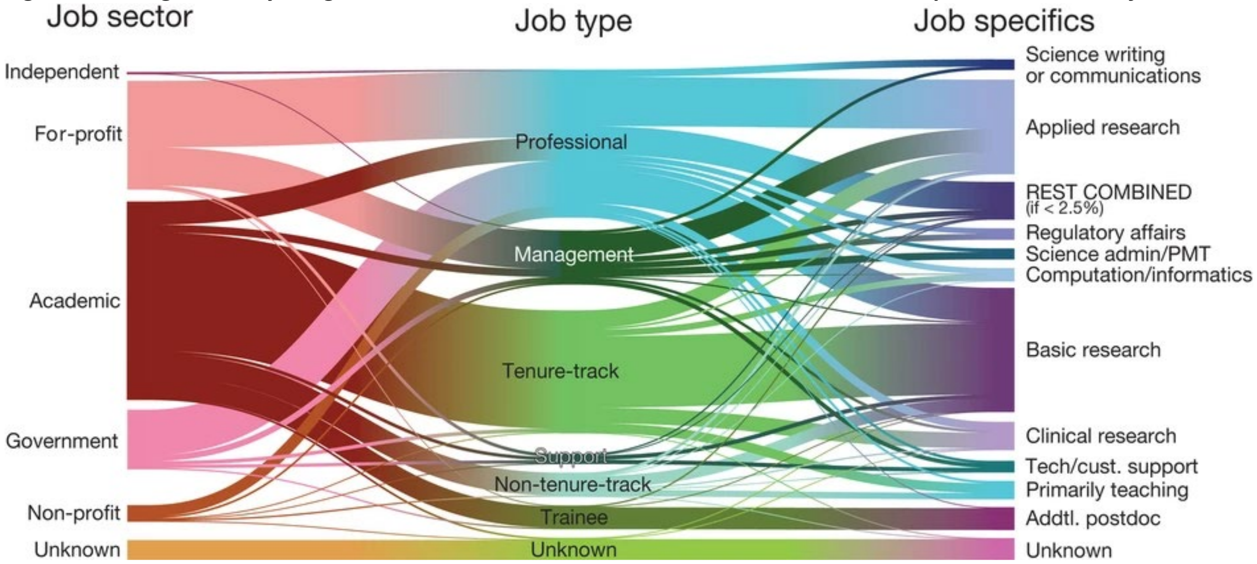

Figure 2 Caption: *A Sankey diagram shows the relationship between three tiers of a taxonomy from postdoctoral alumni at the National Institute of Environmental Health Sciences. Moving from left to right across the diagram, one can see, for example, that the majority of those who enter academia do so as tenure-track faculty. Conversely, it is apparent that most who enter tenure-track faculty are in academia, with a few in the government sector and even fewer in the non-profit sector. Continuing from the middle and moving to the right, it is clear that the majority of those in tenure-track faculty positions are conducting basic research, while a smaller proportion are in applied research, clinical research, or teaching positions. Reading from right to left, it is apparent that most individuals conducting basic research are in tenure-track positions, with a smaller proportion in other types of positions. (Reproduced without changes; https://creativecommons.org/licenses/by/4.0/)* (Xu et al., 2018)

2.3 Directional Chord: A directional chord diagram is similar to the Sankey in that it allows one to analyze the flow between different sets of entities. The entities are displayed around a circle and are connected by arcs. What sets this apart from a typical chord diagram is that it provides directionality, with arrows pointing in the direction of flow, thus making it more apparent to the viewer. In an example from NIEHS, we can see the flow from country of origin into country of employment where arrows point to employment location (Xu et al., 2018) (Fig. 3). Examining Japan, for instance, one can see that nearly all fellows with Japan as their country of origin return to Japan for employment (note the thicker orange arrow/arc). Conversely, a much smaller proportion of fellows from Japan obtain employment within the USA (note the thin orange arrow/arc). At the same time, it is also apparent that there is little flow into employment in Japan from those who originate from countries outside of Japan. In contrast, if we examine China, one can see a thicker purple arrow denoting flow into employment within the USA, with a thinner arrow returning to China. Similar to Japan, there is little flow into employment in China from those with a different country of origin.

Figure 3. Using a Directional Chord Diagram to Illustrate the Relationship Between Country of Origin and Country of Employment.

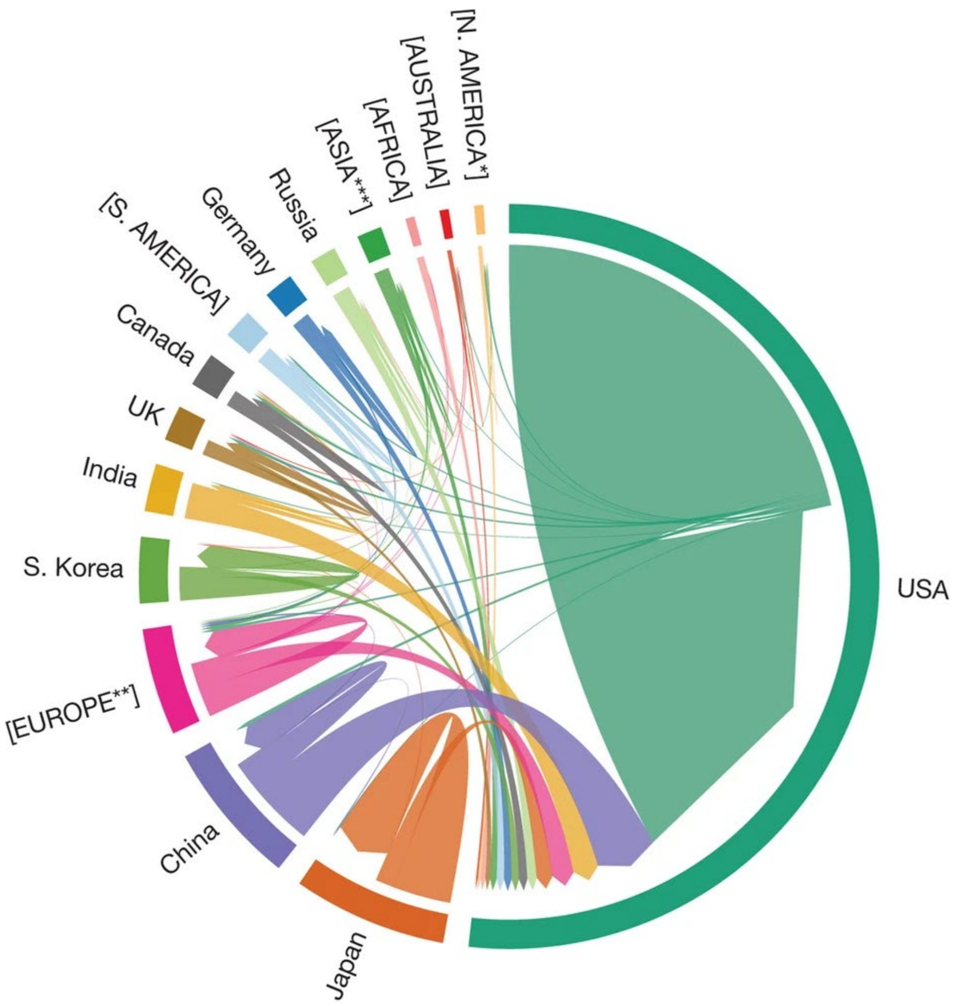

Figure 3 Caption: *This directional chord diagram displays the countries of origin alongside the countries of job location (arrows point to job locations) from postdoctoral alumni at the National Institute of Environmental Health Sciences. Nearly two-thirds of alumni remain in the US after training; fellows from Japan, South Korea, the UK and Germany enter into careers in their home countries more so than fellows from other countries. *North American countries excluding US and Canada; **European countries excluding UK, Germany; ***Asian countries excluding China, Japan, India and South Korea. If there are enough alumni to visualize from an individual country, it is shown in title case. Remaining countries are grouped and depicted by continent in all caps. (Reproduced without changes; https://creativecommons.org/licenses/by/4.0/)* (Xu et al., 2018)

2.4 Stacked Donut or Sunburst: A stacked donut provides a simple way to visualize a snapshot of outcomes, using, for example, the UCOT 2017 three tier taxonomy deployed by Wayne State (Mathur et al., 2018) (Fig. 4). The stacked donut typically consists of concentric circles; in this example, three circles are stacked, with each representing a different point in time from when graduates received their doctoral degrees. The inner circles represent those 0-5 years from receiving their degree; the middle circles represent those 6-10 years out, and the outermost circles represent those 11-15 years from receiving their degree. Career outcomes for each tier of the UCOT 2017 taxonomy are shown, with the top plot representing the employment sector (Tier 1), the middle plot representing the career type (Tier 2), and the bottom plot representing job function (Tier 3). Upon examining the plots, it is readily apparent that the proportion of individuals engaged in further education or training (see Tier 2) declines sharply the further out one is from receiving their degree; likewise, the proportion entering into either faculty or group leader positions (see Tier 3) significantly increases the further out one is from receiving their doctoral degree.

Figure 4. Using Stacked Donut Plots to Illustrate Career Outcomes from Three Taxonomic Tiers at Different Points in Time.

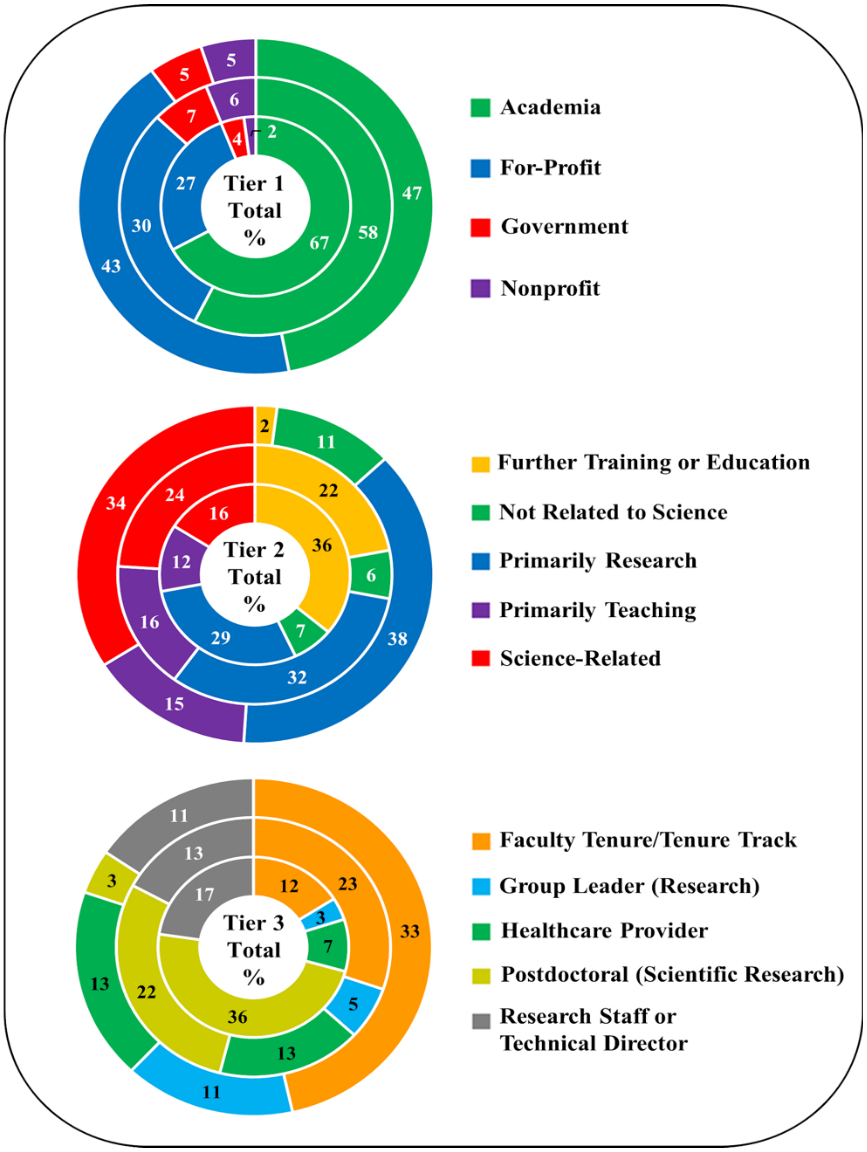

Figure 4 Caption: *These stacked donut plots depict the career outcomes of Wayne State University's biomedical doctoral alumni at different points in time after receiving their degree (either 0–5 years, inner circle; 6–10 years, middle circle; or 11–15 years, outer circle). The top plot represents outcomes of alumni by employment sector (tier 1); the middle plot represents outcomes by career type (tier 2), and the bottom plot represents outcomes by job function (tier 3). (Reproduced without changes; https://creativecommons.org/licenses/by/4.0/)* (Mathur et al., 2018)

2.5 Bubble Plot & Heatmap: A bubble diagram is a powerful visualization tool for multiple variables simultaneously. When overlaid with a heatmap, one can add even greater dimensionality to the data. As shown in an example from NIEHS, career outcomes are separated by country of origin, job type (and relative percentage within that job type), as well as time in postdoctoral position (Xu et al., 2018) (Fig. 5). If we compare the professional staff positions as an example, it is apparent that the greatest proportion of U.S. fellows enter into these types of positions. Examining mean training time shows that they spend on average between 30-40 months in training. If we compare this to fellows from Japan, stark differences emerge—for example, it is clear that fewer fellows enter into professional staff positions relative to the population of fellows from Japan. At the same time, it is also clear that the training time is significantly greater (approaching 60 months) for those fellows from Japan who enter into professional staff positions. While a lot of stories can be told with this type of visualization, one limitation is that it is difficult to discern differences in bubble size, thus restricting one's ability to accurately estimate true percentages. Furthermore, the high degree of data dimensionality could confuse the casual reader. Figure 6 shows another example of a bubble plot reproduced with

permission from the American Historical Association's project to identify where historians work[45]. This plot is more straightforward in that it depicts the relative proportion of alumni entering into careers classified by SOC codes. An interactive version of this plot can be found on the AHA website[46].

Figure 5. Using a Bubble Plot with an Overlaid Heatmap to Illustrate Career Outcome Differences by Country of Origin, Job Type, and Training Time.

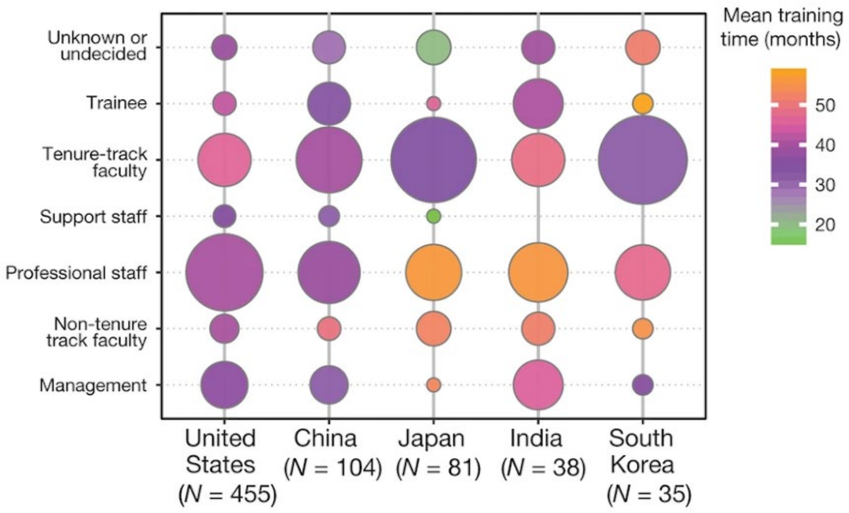

Figure 5 Caption: *A bubble plot showcasing career outcomes from the National Institute of Environmental Health Sciences postdoctoral alumni illustrates how training times vary for those of different countries of origin entering into different job types. The bubble plots illustrate that U.S. alumni enter into professional staff roles at a proportionally higher rate than those from other countries. Alumni from Japan and South Korea are more likely to enter into tenure-track faculty positions than those from other countries. When viewing the heatmap, it becomes apparent that alumni from Japan and India who enter into professional staff positions spend more time in training than those from other countries entering into different job types. (Reproduced without changes; https://creativecommons.org/licenses/by/4.0/)* (Xu et al., 2018)

[45] https://www.historians.org/wherehistorianswork
[46] https://www.historians.org/wherehistorianswork

Figure 6. Using a Bubble Plot to Illustrate the Proportion of Individuals Entering into Careers Classified by SOC Codes.

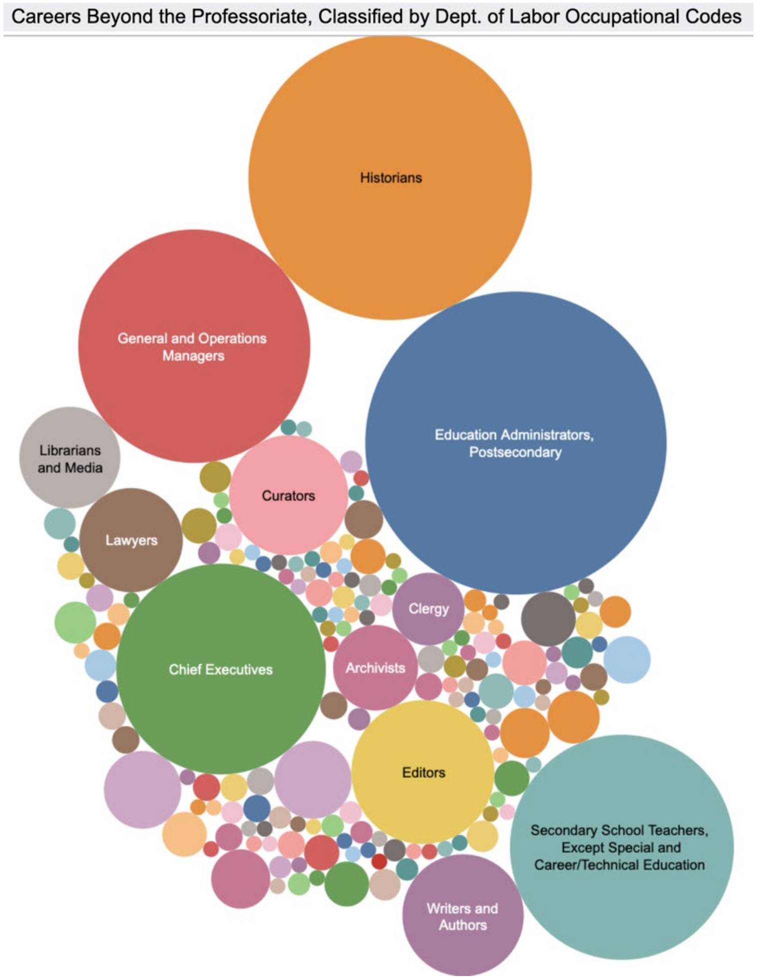

Figure 6 Caption: *The American Historical Association determined the career outcomes of historians and classified them by SOC codes. Their outcomes are depicted in the form of a bubble plot which illustrates the relative proportion entering into different career paths, with the two largest paths being Postsecondary Education Administrators and Historians. Reproduced with permission from AHA[47].*

2.6 Two-way Table: A two-way table, or contingency table, allows one to visualize the relationship between two sets of categorical variables. In an example from Georgetown University depicting the outcomes of those who earned master's degrees, one can view the relative proportion of graduates who enter into different sectors, or job functions, as it relates to their job type/industry (Bhombe et al., 2019) (Fig. 7). From this visualization, it is apparent that those entering into academia are primarily conducting research or are in education. Those who enter BioPharma are most likely conducting research, while others may be engaged in regulatory affairs or marketing/communications. Another example of a two-way table can be found in a study from the University of California-San Francisco in an analysis of their postdoctoral alumni outcomes (Silva et al., 2016).

[47] https://www.historians.org/wherehistorianswork

Figure 7. Using a Two-Way Table to Visualize the Career Outcome Relationship Between Sector and Industry.

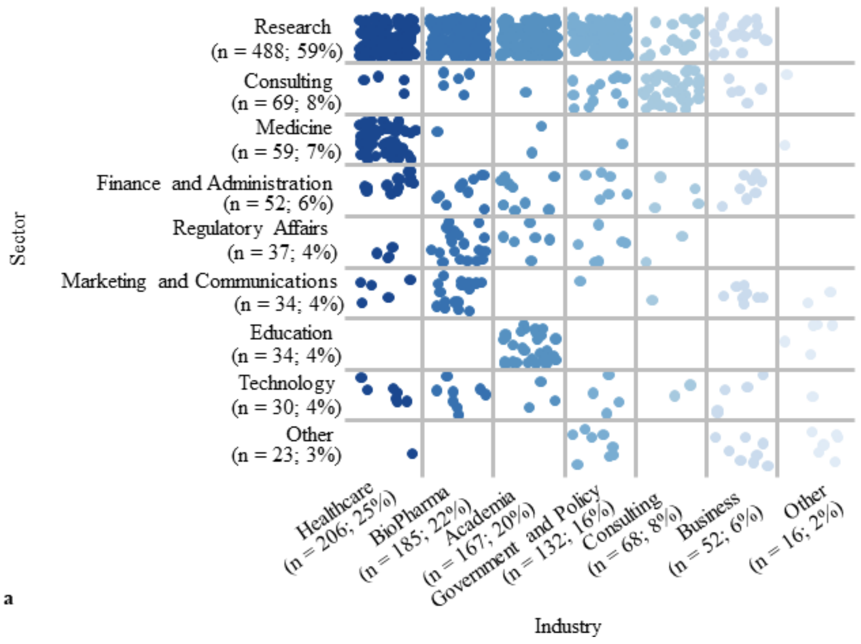

Figure 7 Caption: *A two-way table was used to illustrate the first destination career outcomes of 829 master's graduates from Georgetown University within each sector (job function) and industry (job type). An individual graduate is represented as a dot, with the dots color-coded by industry. It is apparent that most graduates are engaged in research in either the healthcare, BioPharma, academia or government and policy industry. (Reproduced without changes; https://creativecommons.org/licenses/by/4.0/)* (Bhombe et al., 2019)

2.7 Waffle Chart and Small Multiples: A waffle chart allows one to show how the relative proportion of a selected career outcome relates to the whole. The waffle is depicted in the form of a square grid, with the colored areas of the grid representing the data. An excellent example can be found in the National Science Board (NSB) infographic[48] which showcases employment sector data of science, engineering, and health (SEH) doctorates. The data are further represented as small multiples, which is a series of graphs shown together with the same axes and scale allowing for direct comparisons; viewers can thus quickly ascertain how employment trends differ amongst those who received their degrees 15+ years ago versus those who received degrees more recently (relative to when the surveys were conducted in either 1993, 2003, or 2013). The infographic makes further use of small multiples in the form of bar charts to illustrate additional details within each employment sector, including gender, race/ethnicity, job satisfaction, job related to degree, and job duties. For instructions on how to create small multiples in platforms such as Tableau or Excel, multiple websites offering guidance are available online[49-50]. Guidance on creating a waffle plot can be found at Depict Data Studio[51] or one can use the waffle plot template from an infographics toolbox from Google[52].

[48] https://www.nsf.gov/nsb/sei/infographic2/?yr=2013&fd=Mathematics%20and%20statistics&cs=ShowGender
[49] https://depictdatastudio.com/data-table-to-small-multiples/
[50] https://www.juiceanalytics.com/writing/better-know-visualization-small-multiples
[51] https://depictdatastudio.com/charts/waffle/
[52] https://docs.google.com/drawings/d/1KlcdyB3Pkl3NFH7XyO83yPOBKSXcc7km-4k4shgUlq0/edit?ntd=1

Figure 8. Using Waffle Plots and Small Multiples to Illustrate Career Outcomes and Gender Differences at Different Time Points

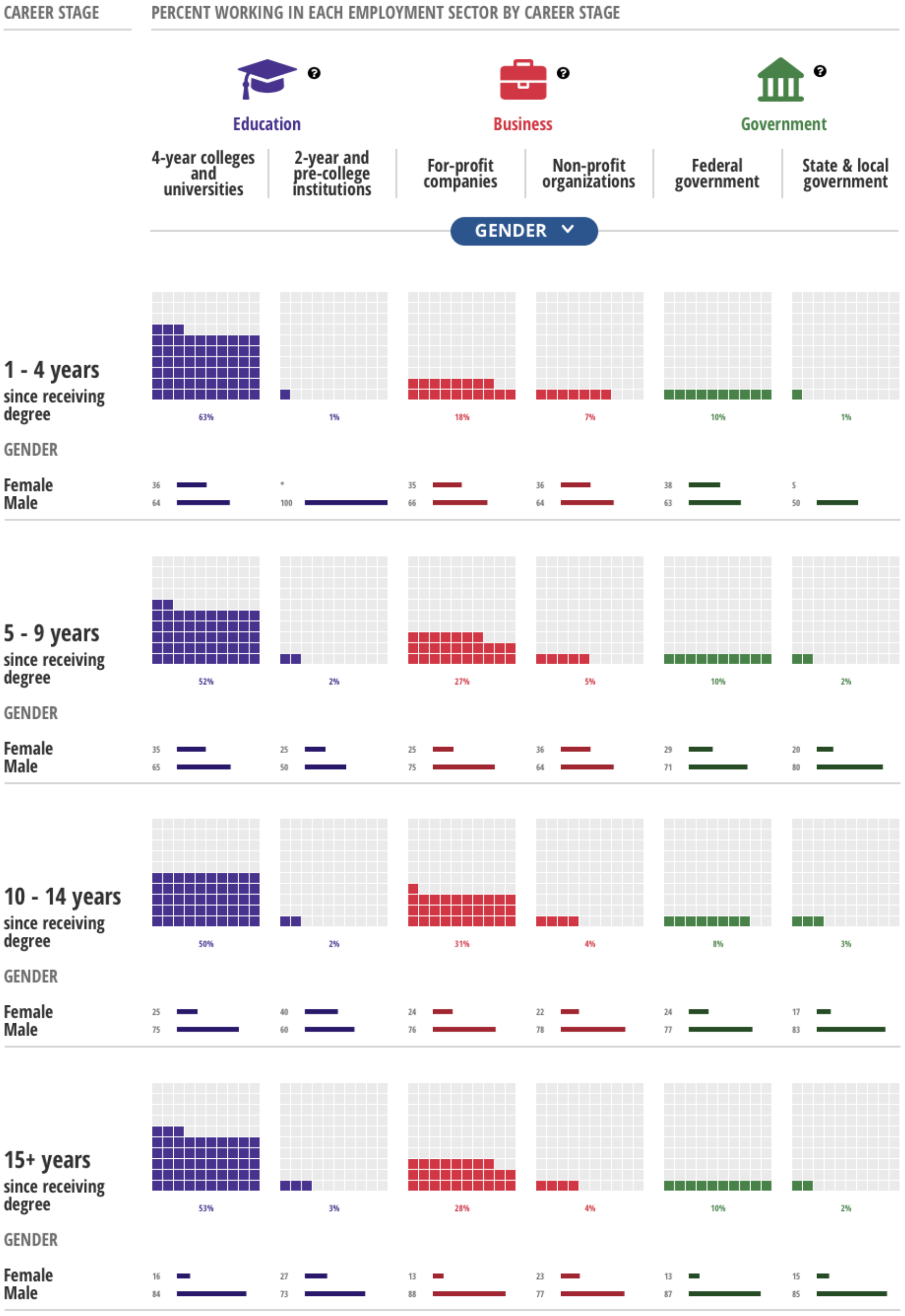

Figure 8 Caption: *The National Science Board used waffle plots used to showcase the relative proportion of science, engineering, and health doctorate (SEH) graduates who entered into different employment sectors relative to how long ago they received their doctoral degree. There is little difference across the various time points, with most entering into 4-year colleges and universities regardless of whether they were 1-4 or 15+ years from receiving their degree. Small multiples represent the relative proportion of males and females comprising the workforce across the different employment sectors and time points. It is apparent that those 15+ years from receiving their degree are largely male. Reproduced with permission from the National Science Board.*

Table 1: Comparison of Platforms & Visualization Types.

Platform or Vizualization Type	Cost	Time/FTE/Training investment level	Skill Level (Advanced, Intermediate, Novice)	Strengths	Points to Consider	Selected Examples
**Tableau**	+	+	Intermediate	Easier to create a dashboard by selecting options & less programming literacy required	Less control of data presentation	U Toronto, Stanford, Michigan, etc.
**R (& NIEHS dashboard)**	Free	+++	Advanced	More control of how to present data (predeveloped dashboard can be modified for institutions)	Need programming literacy	NIEHS
**Excel**	+	+	Novice	Ease of use for those without much technical experience or resources	Limited visualization options, not much room for customization, not interactive, and low quality	OHSU
**Career Services Management Platforms**	+++	+	Novice	Optimized for those without extensive data analysis experience; systems automate many of the visualizations; some level of customization available	Limited to the visualization options included in the systems with not as much room for customization	Symplicity, Handshake, 12Twenty
**Microsoft PowerBI**	+	++	Advanced	Easy data sharing and intuitive user-interface; can customize visualizations; visualizations are interactive	Not as widely used in the field and may require additional training; may not contain all visualization options desired	Wayne State, University of Texas System, Weill Cornell Medicine, NACE
**Data Analytics Software (Prism, SPSS, SAS)**	Varies (+, +++)	Varies (+, +++)	Advanced	Customizable options for analysis (SPSS, SAS, Prism) and data vis (SAS, Prism)	Plug and play dropdown menus, relatively easy to learn (Prism & SPSS); versatile options by checkbox and extensive tutorials (Prism); coding more time intensive but more customizable (SAS, SPSS)	NIH BEST

**Institution-Specific**	+++	+++	Advanced	Fully customizable and can make use of available Google or other libraries	Requires programming and software development knowledge; likely requires partnering with groups across one's institution	Boston University, Clemson, UBC
**Divergent Stacked Bars**		+	Novice	Readily see trends in outcomes that decrease or increase over time at diverging point	More difficult to ascertain changes in bars not precisely at the divergent point; care should be made to explain the X-axis	OHSU, Scripps, NIEHS
**Sankey**		++	Advanced	Show broad patterns of complex data all at once	Sector flow and time flow can be misinterpreted	OHSU, NIEHS, Wayne State, Michigan, UBC, Stanford
**Directional Chord/Chord**		+	Advanced	Show broad patterns of complex data all at once	Not always immediately obvious; need arrows	NIEHS, Williams College
**Stacked Donut/ Sunburst**		+	Intermediate	Readily see differences in career outcomes across different year-cohorts	Obligate smaller interior donut and larger exterior donut could obfuscate some comparisons	Wayne State
**Bubble (+Heatmap)**		++	Advanced	Compare outcomes of different cohorts simultaneously; overlay with additional dimensions	Relative bubble size; overestimation	NIEHS, American Historical Association
**Two-way Table**		+	Novice	Compare two sets of categorical variables; illustrate with values, heatmap or dots	Color coding by variables could be mistaken for a heatmap or could obfuscate comparisons	Georgetown University, UCSF
**Waffle Plot**		+	Novice	Select outcomes can be clearly highlighted as progress towards 100%	Too many outcomes on the same plot could become complicated	National Science Board
**Small Multiples**		++	Intermediate	Powerful method allowing for a quick assessment of overall patterns in data	If multiples are too small they become illegible	National Science Board

Table 1 Caption: Adapted from AAMC GREAT 2018 project (Baas et al., 2018).
